# Direct-inject liquid-chromatography enables an autonomous continuous preferential crystallization platform

**DOI:** 10.1039/d6sc05647d

**Published:** 2026-09-28

**Authors:** Joshua S. Derasp, Felix Painsecq, Wenyu Zhang, Matthew E. Reish, Paloma Prieto, Jason E. Hein

**Affiliations:** a Department of Chemistry, The University of British Columbia Vancouver British Columbia V6T 1Z1 Canada jason.hein@ubc.ca; b Acceleration Consortium, The University of Toronto Toronto Ontario M5G 1X6 Canada; c Université de Rouen Normandie Place Emile Blondel 76821 Mont-Saint-Aignan France; d Department of Chemistry, University of Bergen Bergen N-5020 Norway

## Abstract

Access to both enantiomers of chiral building blocks remains a fundamental challenge in chemical synthesis, particularly for scalable applications in pharmaceuticals, agrochemicals, and materials development. Continuous preferential crystallization (CPC) offers a promising route to obtain enantiomerically enriched compounds from racemic mixtures without the use of chiral auxiliaries. However, its widespread adoption is hindered by the need for precise supersaturation control mediated by real-time process analytical technologies capable of monitoring enantiomeric ratio. Here, we report the development of an autonomous CPC platform enabled by online chiral high-performance liquid chromatography. Using our Direct Inject Liquid Chromatography (DILC) module, we continuously monitor the crystallization process and implement feedback control that initiates temperature-based dissolution in response to early detection of undesired enantiomer accumulation. The trigger threshold is set from the measured noise in the baseline signal, so the controller responds to signal breakthrough rather than the scatter in the measurement, allowing it to run for days without supervision. Applied to the CPC of allylalanine monohydrate, a conglomerate-forming α-amino acid, this system achieved multi-day operation and gram-scale production of enantiopure material with minimal supervision. The platform establishes a generalizable strategy for real-time control of enantioselective crystallizations and opens new avenues for self-driving purification technologies.

## Abstract 2

Sampling the slurry directly, online chiral HPLC detects the unwanted enantiomer within the growing solid and triggers a thermal reset that purges it, enabling five days of autonomous preferential crystallization of both enantiomers.

## Graphical abstract

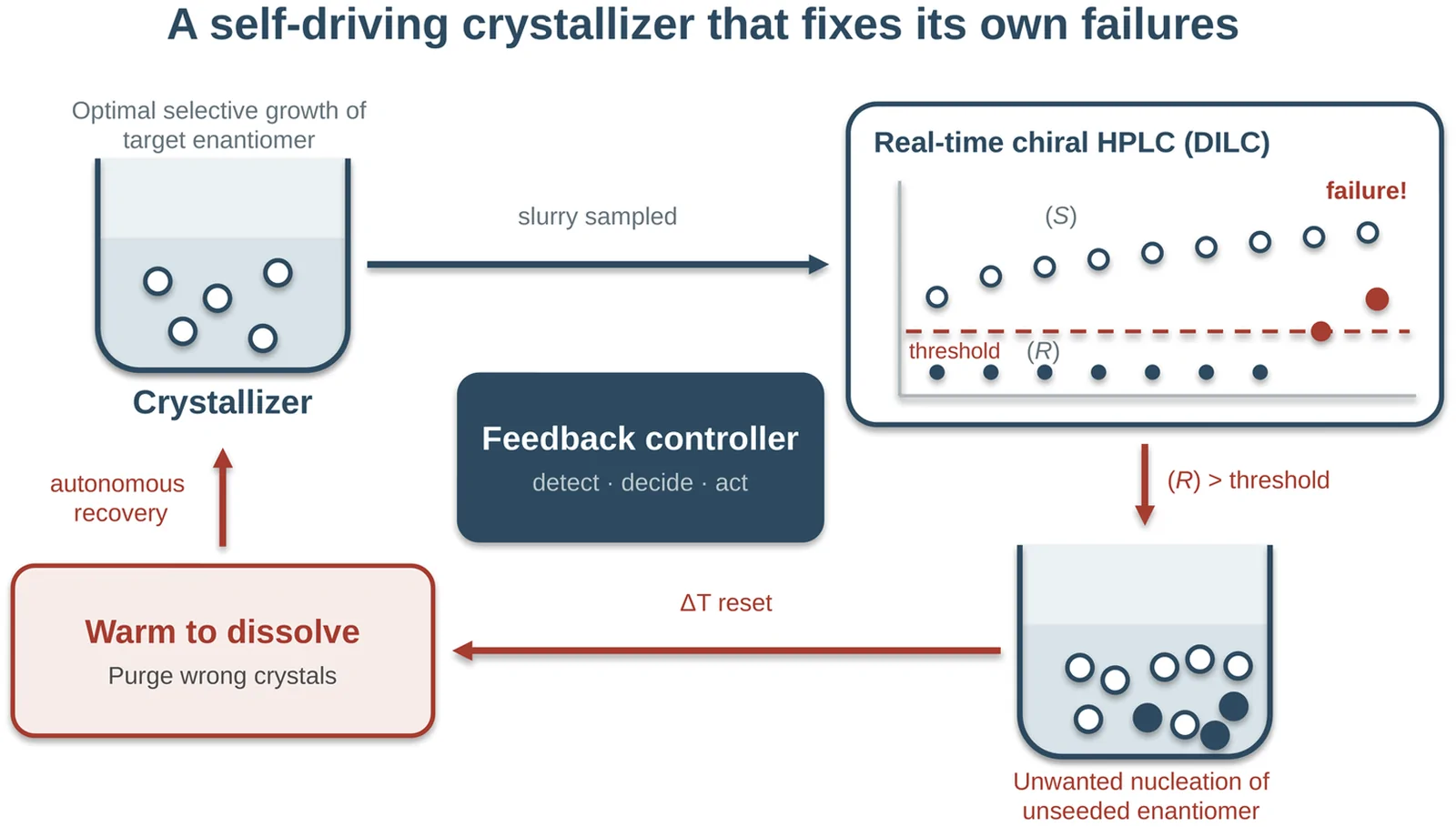

## Introduction

The global demand for enantiomerically pure compounds continues to accelerate, driven by their essential roles in pharmaceuticals, agrochemicals, diagnostics, and materials science. The chiral chemical market was valued at USD 77.4 billion in 2024 and is projected to exceed USD 125 billion by 2030, reflecting both commercial growth and increasing regulatory pressure for stereochemically-defined active ingredients.^1^ Efficient and scalable access to both enantiomers of a target molecule remains a central challenge in chemical manufacturing.^2^ Among the range of technologies developed to address this need, crystallization offers a particularly attractive solution due to its inherent scalability, operational simplicity, and compatibility with solvent-based processes.^3^ Unlike asymmetric catalysis, which typically delivers a single stereoisomer and often requires expensive chiral ligands or reagents, crystallization-based approaches can resolve racemates directly into their individual enantiomers, often without chemical transformation, making them especially well-suited to produce chiral building blocks and bulk intermediates.

α,α-Dialkyl amino acids are an important class of chiral fine chemicals, with applications spanning natural products,^13^ organocatalysts,^14^ radiotracers,^15^ peptidomimetics,^16^ and peptide stapling.^17^ Accessing them in high enantiopurity on scale remains challenging and costly, even with modern asymmetric catalysis.^18^ α-Allylalanine is a particularly attractive member of this family, because the terminal alkene is an orthogonal handle for late-stage diversification that allows a single enantiopure batch to be funnelled into a range of α,α-disubstituted amino acids without revisiting the asymmetric step. This versatility is well documented. The alkene has been engaged in stereoselective halolactonization to constrained γ-lactones and proline analogues,^22^ in hydroboration and radiofluorination to deliver an amino-acid positron-emission-tomography tracer,^15^ and in cross- and ring-closing metathesis to furnish acyclic and cyclic quaternary amino-acid derivatives.^23,24^ The same α-methyl, α-alkenyl motif underpins all-hydrocarbon peptide stapling, in which ring-closing metathesis of two such residues rigidifies therapeutic α-helices.^17,25^ Demand for these building blocks is reinforced by the resurgence of peptides as a therapeutic modality.^26^ GLP-1 receptor agonists, including semaglutide and the dual GIP/GLP-1 agonist tirzepatide, have transformed the treatment of type-2 diabetes and obesity,^27,28^ and macrocyclic peptides are now reaching oral, traditionally small-molecule indications, as exemplified by the oral PCSK9 inhibitor enlicitide.^29,30^ Such molecules are assembled from non-proteinogenic and α,α-disubstituted amino-acid residues, and their viability depends on the scalable, enantioselective manufacture of these building blocks. Recent biocatalytic-cascade routes to enlicitide illustrate that this manufacturing step is often rate-limiting and value-defining.^31^

We recently reported allylalanine monohydrate as a conglomerate-forming compound.^19^ That study demonstrated minimal mother liquor entrainment, significantly reducing solid-phase contamination and enabling accurate measurement of enantiomeric purity. Despite these favourable attributes, the compound presents crystallization challenges including multiple solid-state polymorphs and a narrow metastable crystallization window, complicating conventional batch processing and highlighting the need for improved process control. As established in this work, the system also departs from ideal conglomerate behaviour under the supersaturation excursions encountered during extended operation, a deviation that we characterise here. Racemic allylalanine monohydrate for this work was prepared in a single step by a Petasis reaction between pyruvic acid, ammonium hydroxide and allylboronic acid pinacol ester in 27% unoptimized yield, in place of the five-step sequence used previously, with full synthetic details and characterization provided in the SI.^19,20^

Preferential crystallization (PC) is well matched for building blocks of this kind, where demand exists for both enantiomers at scale. It enables the selective crystallization of one enantiomer from a supersaturated racemic solution through seeding, capitalizing on a kinetic advantage in secondary nucleation and growth of the desired enantiomer while the unseeded enantiomer remains in solution. In contrast to classical resolution, which relies on the formation of diastereomeric salts through the addition of a resolving agent, PC is operationally simpler and avoids the need for chemical derivatization. Its success, however, depends on the use of conglomerate-forming systems, which are unique chiral molecules that crystallize into enantiomerically pure domains rather than racemic crystals. These systems were long thought to be rare, historically estimated to comprise only 5–10% of racemic compounds.^5^ However, recent reanalysis of the Cambridge Structural Database and related crystallographic surveys suggest that this figure significantly underrepresents the true prevalence. Revised estimates place the lower bound closer to 19%, with many previously misclassified systems being reidentified as conglomerates.^6,7^ This growing recognition expands the accessible chemical space for PC far beyond earlier expectations and suggests that preferential crystallization could serve as a general tool for the scalable, reagent-free separation of enantiomers in both academic and industrial settings.

While preferential crystallization offers a conceptually elegant route to enantiopure compounds, its practical execution remains technically demanding. The success of the process hinges on maintaining a metastable supersaturation regime that favors the growth of the seeded enantiomer while suppressing spontaneous nucleation of the undesired form. Achieving high yield and productivity requires pushing the system toward large enantiomeric imbalances in solution, but doing so also increases the risk of failure through uncontrolled primary nucleation of the wrong enantiomer. Once such an event occurs, it can be irreversible, leading to mixed solid phases and significant erosion of enantiopurity. This inherent tension between productivity and process stability has long limited the broader implementation of PC, particularly in settings where hands-off or scalable operation is desired.

To address these challenges, significant progress has been made in developing advanced resolution protocols that strategically manage supersaturation dynamics. One well-developed example is auto-seeded polythermic programmed preferential crystallization (AS3PC), in which a programmed cooling profile drives a racemic, slightly enantio-enriched solution through self-seeding and selective growth of the seeded enantiomer, alternated with controlled dissolution to recover the antipode.^32^ However, the practical implementation of AS3PC and related protocols remains highly manual and requires deep process knowledge. Cycle timing, seed quality, and temperature control must be precisely managed to avoid uncontrolled nucleation or incomplete resolution. Even small deviations in execution can result in batch failure or require restart. These constraints pose significant barriers to routine use, particularly at scale or in workflows that demand reproducibility, low operator involvement, or integration into continuous manufacturing settings.

To overcome the labor intensity and fragility of batch-mode strategies, continuous preferential crystallization (CPC) has emerged as a promising evolution of preferential resolution.^4^ By spatially separating the crystallization and dissolution zones and continuously recirculating solution between them, CPC architectures allow for steady-state operation, improved throughput, and reduced reliance on manual intervention. However, CPC inherits the same failure mode as batch processes. Spontaneous primary nucleation of the undesired enantiomer becomes more likely over the extended runtimes that continuous operation requires, and enantiomeric enrichment becomes inconsistent as a result. Without process monitoring and control, these failures go undetected until offline analysis reveals compromised purity, by which point material has been lost and the run interrupted.

CPC adoption is limited by the lack of a real-time, enantioselective process analytical technology (PAT) that reports both chiral purity and solution concentration (Fig. 1A). The techniques routinely used in crystallization development, such as focused beam reflectance measurement (FBRM), Fourier transform infrared spectroscopy (FTIR) and Raman spectroscopy, return particle size or bulk solute concentration and cannot resolve enantiomeric composition. Online polarimetry has been explored as a chiral PAT for CPC, but its low sensitivity, susceptibility to temperature and solvent effects, and lack of structural specificity limit its reliability and scope.^8^ As a result, CPC processes remain heavily reliant on periodic manual sampling and offline chiral HPLC analysis, making them incompatible with the autonomy, responsiveness, and throughput demanded by modern chemical manufacturing.^9,10^

Recently, our lab developed the Direct Inject Liquid Chromatography (DILC), which provide solution-phase concentration data in real time, enabling the rapid dissection and optimization of complex classical resolutions and second-order asymmetric transformations.^11^ The DILC has proven a robust platform capable of reliable automated sampling even in difficult heterogeneous settings.^12^ In this work, we present a fully autonomous CPC platform enabled by real-time chiral high-performance liquid chromatography using a DILC module (Fig. 1B). This system samples the heterogeneous mixture directly from the crystallizer and dissolves the withdrawn aliquot in line before injection onto a chiral stationary phase, returning both enantiomeric ratio and solute concentration at high sensitivity and resolution without user intervention. Accumulation of the undesired enantiomer in the sampled solid is the earliest indicator of process failure, and the platform tracks it continuously. When that signal exceeds a set threshold, a feedback algorithm raises the crystallizer temperature to dissolve the offending phase selectively, restoring enantiopurity before productive operation resumes. The correction is sized to purge the nucleation event without overshooting into yield loss. Using allylalanine monohydrate as a model conglomerate, we show that the platform sustains multi-day CPC with minimal human input and delivers gram-scale quantities of highly enantioenriched product.

**Fig. 1:**
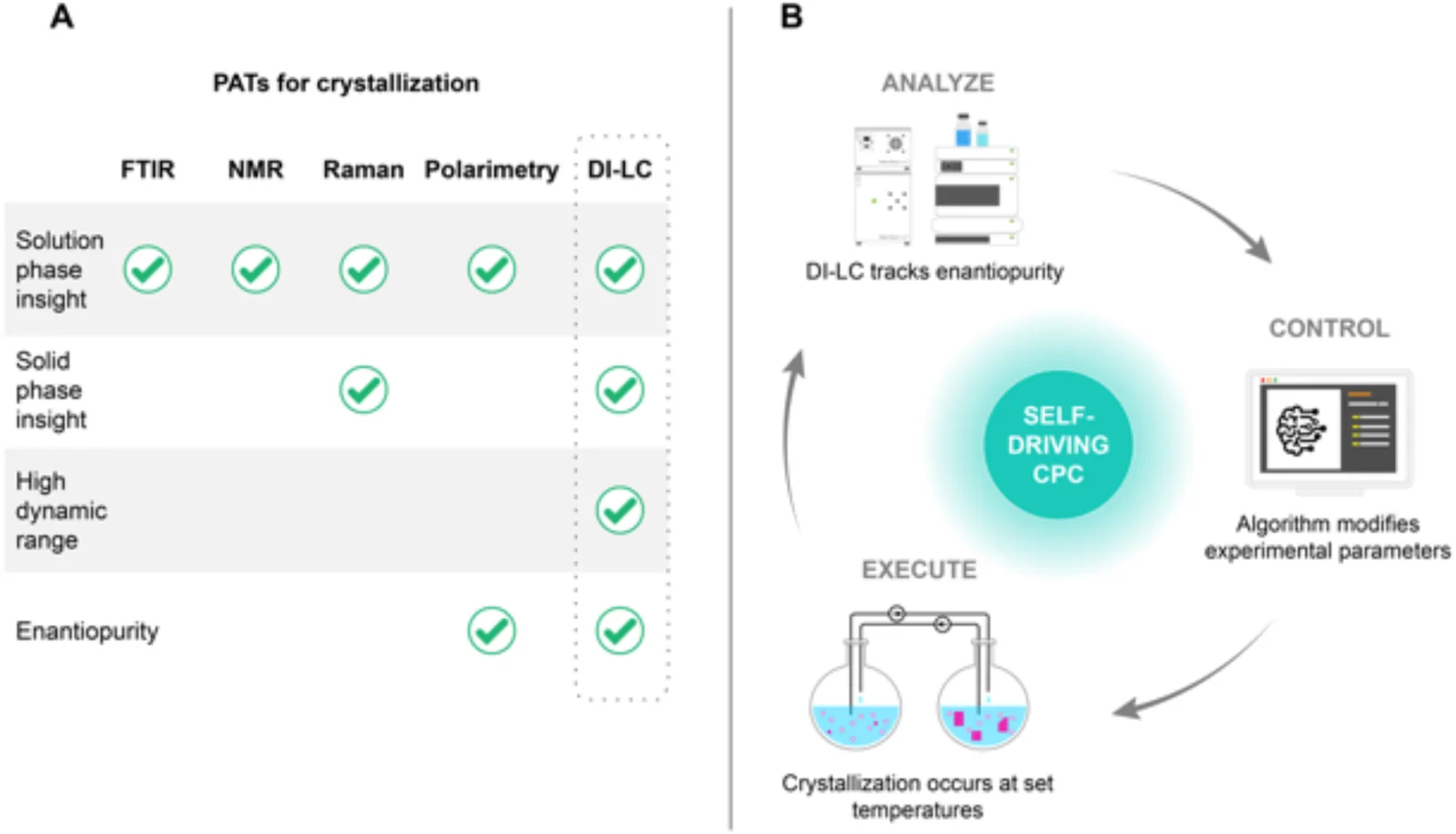
(A) Overview of common PAT techniques used to monitor crystallizations. (B) This work, combining the DILC with a feedback control algorithm to drive CPCs to completion in a self-driven fashion.

## Results and discussion

### Coupled preferential crystallization platform design

There are several possible configurations for conducting CPCs; we chose to implement a coupled preferential crystallization and dissolution (CPCD) system (Fig. 2). This setup involves two interconnected reactors: one acts as the “dissolver” and the other as the “crystallizer”. The dissolver is charged with a suspension of racemic material while the crystallizer contains saturated, solid-free supernatant continuously pumped back and forth from the dissolver. The crystallizer is then seeded with enantiopure seed crystal, and controlled secondary nucleation achieves selective crystallization of the desired enantiomer within the crystallizer. This depletes its concentration in the solution phase, which is then replenished upon return to the dissolver. In our case, a temperature gradient was targeted to serve as the driving force. The dissolver temperature is set to control the saturation concentration of the system, while the crystallizer temperature is set slightly lower, creating a supersaturated environment within the crystallizer that, in addition to the enantiopure seeds, drives stereoselective crystallization.

**Fig. 2:**
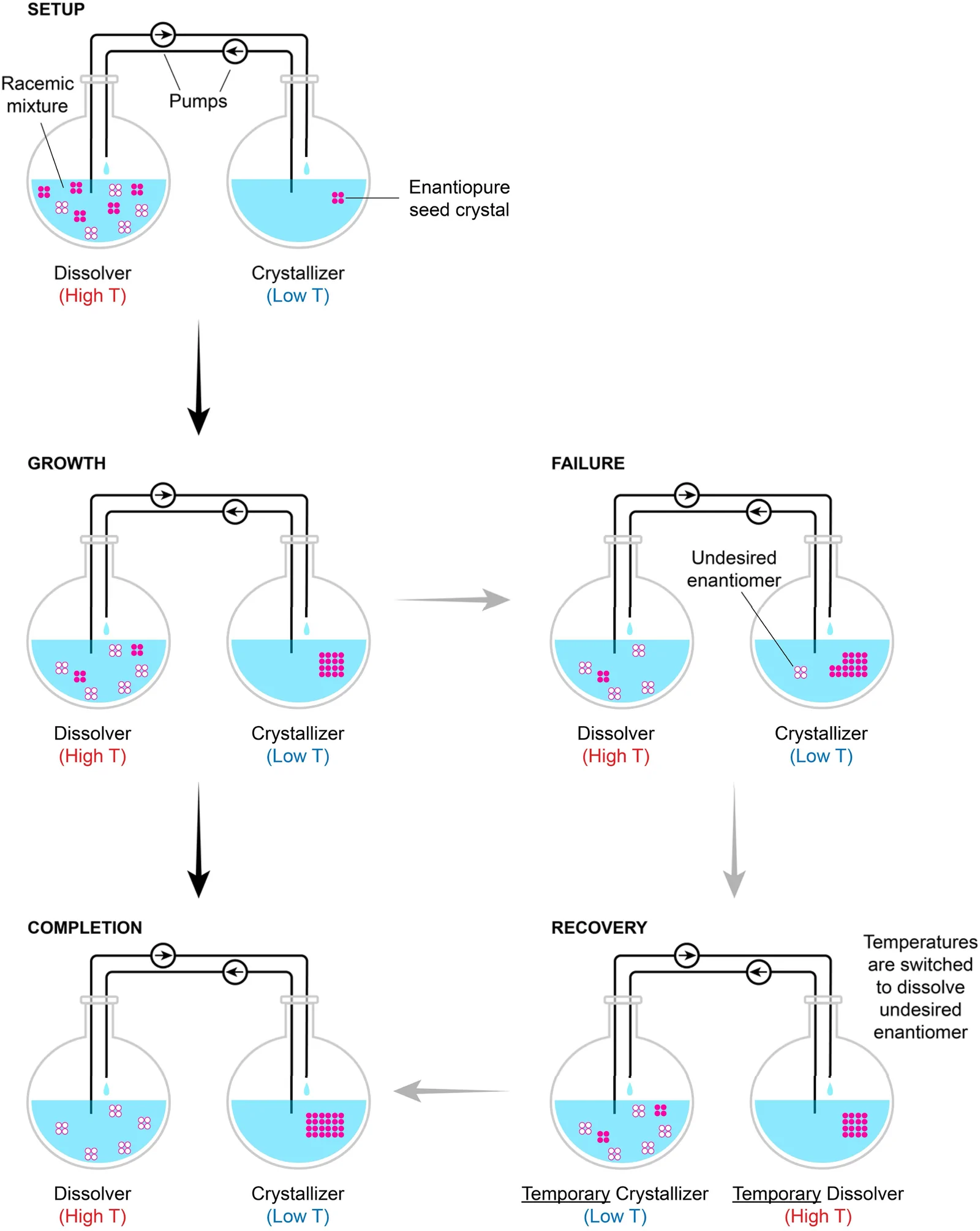
Depiction of the CPC-D setup along with crystal growth, a recoverable failure, its recovery, and process completion.

The central vulnerability of any CPC is thermodynamic rather than operational. Because the feedstock is racemic, the undesired enantiomer is always present in solution, and under crystallization conditions its spontaneous primary nucleation is, given sufficient time, inevitable. In conventional batch preferential crystallization such an event is effectively terminal, forcing the run to be discarded. A defining advantage of the coupled crystallizer-dissolver architecture is that it renders this failure reversible (Fig. 2). Because the two enantiomers of a conglomerate are isoenergetic and therefore share an identical solubility, transiently raising the crystallizer above the dissolver temperature dissolves the accreted solid without any need for chemical selectivity. As dissolution proceeds, the small, newly formed population of the undesired enantiomer is exhausted well before the abundant desired phase, selectively purging the contaminant at minimal cost to yield. Returning the crystallizer to its original set point restores supersaturation, and productive growth of the desired enantiomer resumes. In effect, a single, readily actuated variable, the crystallizer temperature, converts an otherwise catastrophic and irreversible event into a controllable, self-correcting excursion, providing precisely the physical handle required for autonomous operation.

We reasoned that the DILC could fill this gap. With the crystallizer held at a constant saturation temperature, the undesired enantiomer signal should stay flat while the desired enantiomer signal climbs as material transfers from the dissolver and accretes as slurry in the crystallizer. A recoverable failure then announces itself as a sustained rise in the undesired signal above its saturation level, and its correction is confirmed when that signal returns to baseline after the temperature excursion purges the contaminant. The DILC therefore reports on both the liquid phase and the growing solid in real time.

### Hardware and software setup

To conduct our CPC, we required precise temperature control over both reactors and therefore employed the Mettler Toledo EasyMax system. Two SF-10 pumps recirculated solid-free supernatant between the two reactors through inline filters, while an evaporative light scattering detector (ELSD) was used for HPLC analysis owing to the low UV activity of allylalanine (Fig. 3).^19^

**Fig. 3:**
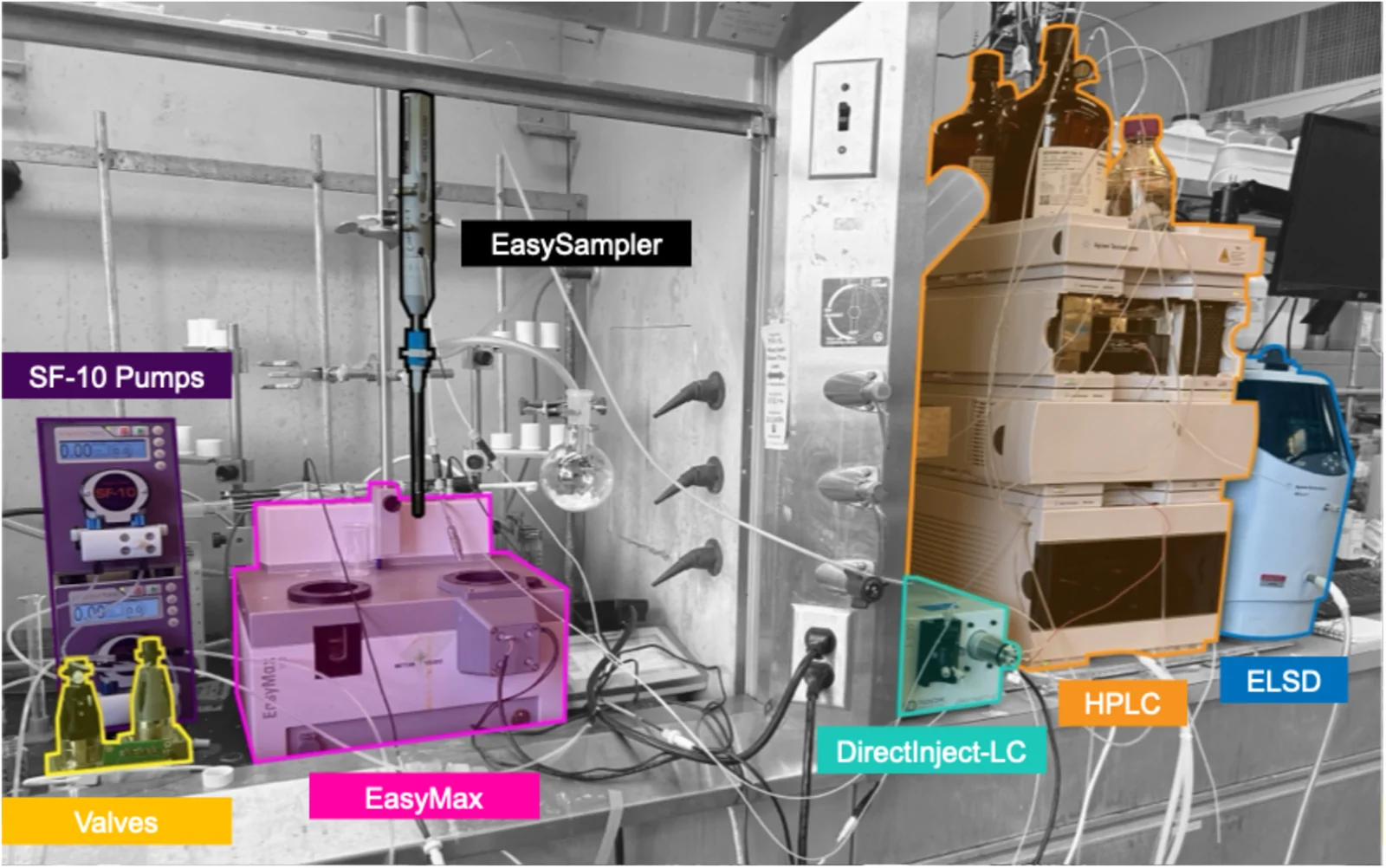
Annotated photograph of the CPC platform. From left to right: SF-10 pumps, two-position four-port valves, EasyMax 102, EasySampler probe, DILC, HPLC, and ELSD.

### A self-flushing reciprocating-flow valve for slurry handling

Sustained operation imposes a demanding fluidic requirement, because the supernatant recirculated between the vessels must remain solid-free while one of them holds an actively crystallizing slurry. Inline filtration is therefore unavoidable, yet drawing supernatant through a frit submerged in a dense slurry is precisely the condition under which filters fail. A particle cake accumulates on the upstream face of the frit and progressively throttles flow until the line occludes and the process stalls. This fouling is one of the principal reasons that continuous slurry recirculation and online slurry sampling remain difficult to sustain without supervision. The problem is compounded here by the pumps, because the SF-10 units are unidirectional and the cake cannot simply be cleared by running the pump in reverse.

We addressed this with a deliberately simple piece of flow hardware. A pair of two-position, four-port valves is placed between the unidirectional pumps and the two reactors (Fig. 4). In one valve position the pumps draw supernatant from the crystallizer through its frit and deliver it to the dissolver. Toggling the valves reroutes the same unidirectional pumps to drive flow in the opposite sense, from the dissolver back to the crystallizer, without reversing the pumps themselves. The consequence for fouling is the central point. On each switch, the frit that had just been acting as the suction inlet, and that had therefore been accumulating a cake, becomes the delivery outlet. Clean supernatant is now pushed backward through that same frit, back-flushing the deposited solids off its face and returning them to the reactor. Filtration duty alternates between the two frits, and each is back-flushed on every cycle before its cake can grow thick enough to restrict flow.

**Fig. 4:**
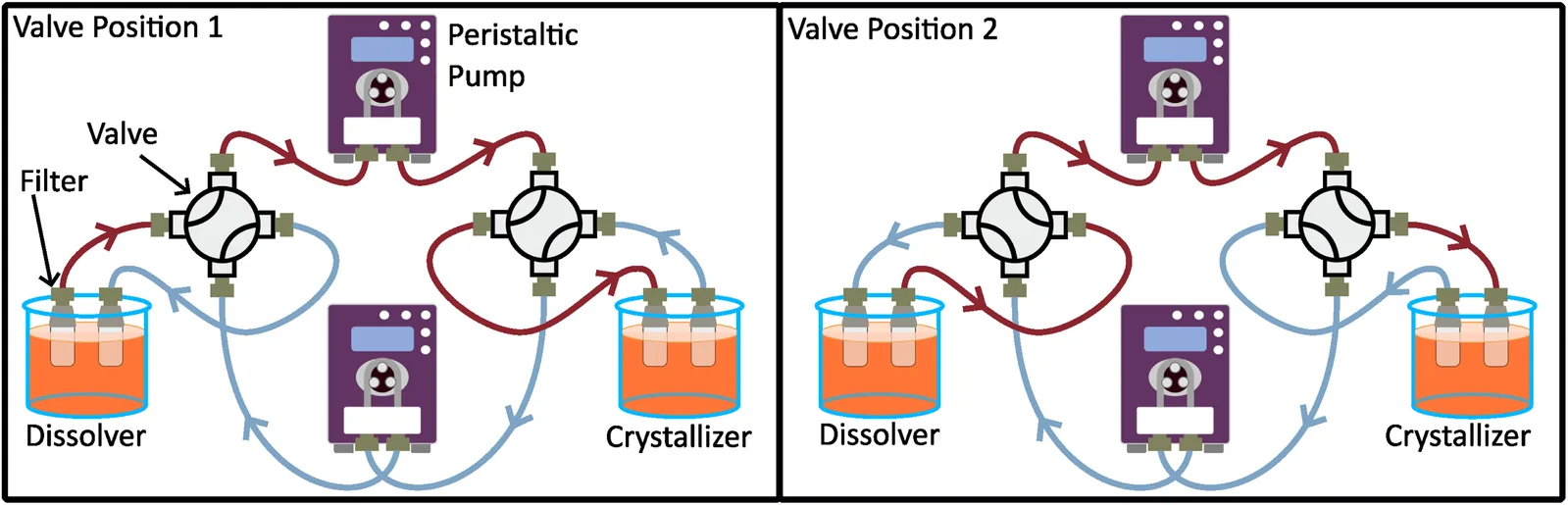
Flow diagram of the valve layout used to reverse flow through the transfer filters. Tubing shown in red always flows left to right and tubing shown in blue always flows right to left; only the segments between the valves and the vessels change direction on switching.

Because the valves toggle automatically on a fixed timer, this self-flushing is built into normal operation and demands no user intervention. In practice it converted filter fouling from a runtime-limiting failure into a non-issue and was a prerequisite for the multi-day, largely unattended campaigns described below. More broadly, reciprocating flow through paired filters provides a low-cost, pump-agnostic route to recirculating or sampling clear liquid from a dense slurry, a recurring obstacle across continuous crystallization and online process analytics. The toggle interval was 15 min; the valve actuation hardware, CAD and firmware files, and a bill of materials are given in Section S3.

### Proof-of-concept: real-time chiral HPLC monitoring for the resolution of allylalanine

The analytical information provided by the DILC depends on whether the sampled stream consists solely of the mother liquor or contains suspended crystalline material. For crystal free supernatant samples, both the (*R*) and (*S*) chromatographic signals represent the dissolved concentrations of the respective enantiomers. During preferential crystallization, the dissolved concentrations remain equal at saturation, such that correlated changes in both signals reflect changes in equilibrium solubility arising from temperature or composition.^33,34^

When a slurry is sampled, the DILC quantifies both the dissolved and suspended solid fractions within the injected sample.^12,35^ Under these conditions, each enantiomeric signal represents the sum of its dissolved concentration and any entrained crystalline material. Consequently, an increase in the (*S*) signal while the (*R*) signal remains fixed at saturation indicates selective growth of the desired (*S*) crystalline phase. In contrast, any increase in the (*R*) signal above its saturation baseline cannot originate from the solution phase, because the dissolved concentration is fixed by the solubility equilibrium, and therefore reports the appearance of (*R*)-allylalanine within the sampled solid.

Because the solution feeding the crystallizer (from the dissolver) is racemic as it is in equilibrium with a conglomerate feed, the liquid contributes equally to the (*R*) and (*S*) signals, and is fixed by the solubility. Any departure from the racemic ratio is due to enrichment in the solid captured in the crystallizer, measured against a solution-phase background acting as its own internal reference. Therefore, slurry sampling by the DILC returns the enrichment in e.r. of the crystalline product in the crystallizer. What the signal does not resolve is the origin of the counter-enantiomer: (*R*) present as discrete crystals from primary or secondary nucleation and (*R*) incorporated into the lattice of the growing (*S*)-rich phase both raise signal from the (*R*) enantiomer in the crystallizer, and both erode product enantiopurity. The controller threshold is an operational trigger for corrective action rather than an assignment of mechanism, and it is effective whichever pathway dominates. It is also this measurement that later reveals allylalanine monohydrate to depart from ideal conglomerate behaviour over an extended campaign.

This is a fundamentally different measurement from the one made by any established chiral PAT. Online optical rotation is the standard chiral sensor for closed-loop control of preferential crystallization.^8^ However, optical rotation would be blind to the failure we are targeting as there is no liquid-phase enrichment to detect. This is because the crystallizer contents remain in contact with a saturated racemic solid, so the solution composition is set by solubility regardless of how much enriched solid is suspended in it. This is a thermodynamic constraint rather than a limit of polarimeter sensitivity, and it applies to any technique that samples clarified mother liquor. The DILC's ability to capture and interrogate the full slurry makes it uniquely able to provide a self-correction signal for this process.

To demonstrate these analytical characteristics, a representative preferential crystallization experiment was performed (Fig. 5). Racemic allylalanine was equilibrated overnight at 20 °C within the dissolver vessel before crystal free mother liquor was continuously circulated to the crystallizer through the reciprocating filtration system, maintaining particle free transfer throughout the experiment. The crystallizer temperature was subsequently reduced to 17 °C to establish a modest supersaturation. Initial DILC measurements produced identical (*R*) and (*S*) responses corresponding to the equilibrium saturated solution, establishing the analytical baseline shown in Fig. 5.

**Fig. 5:**
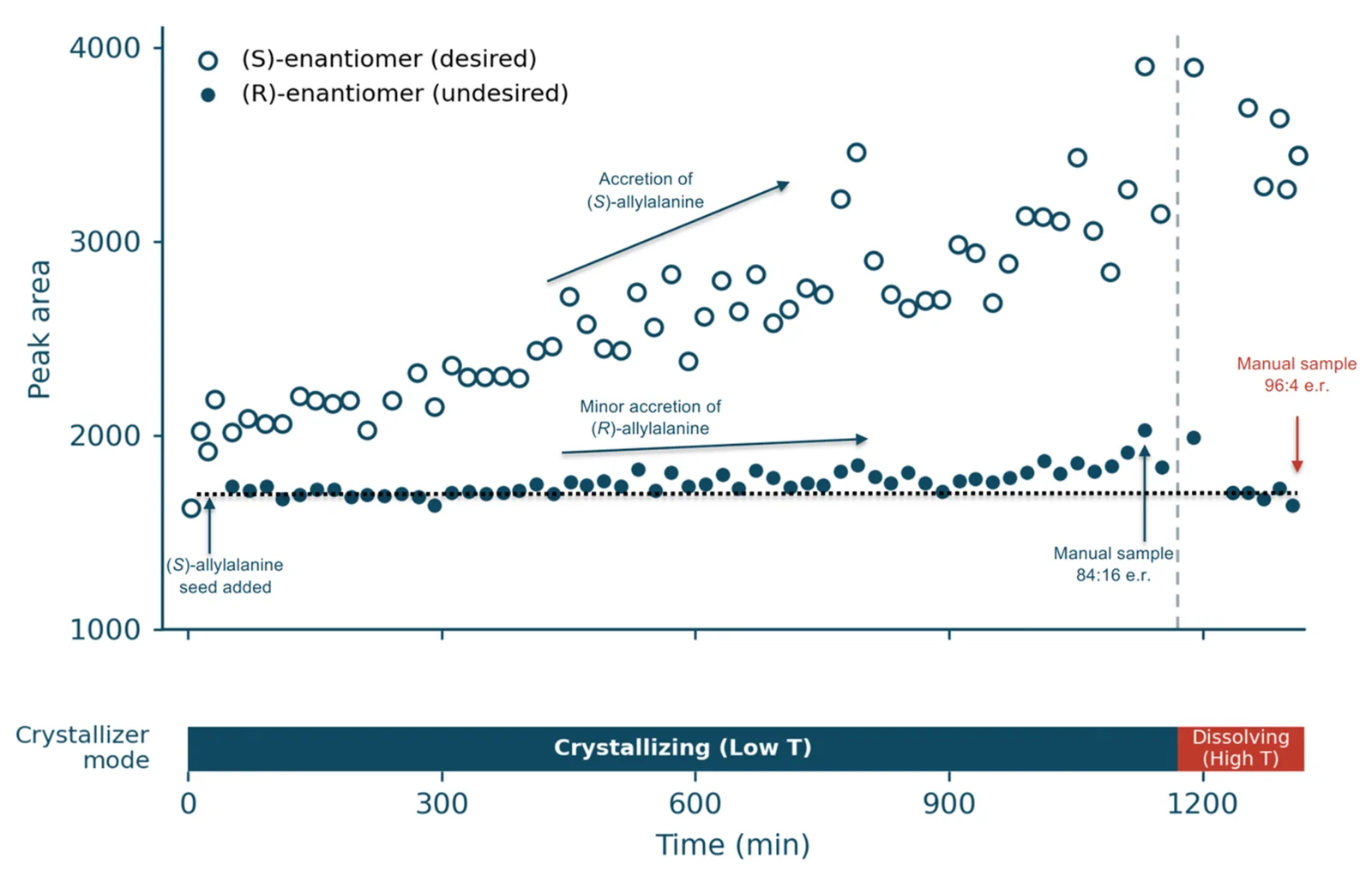
Real-time DILC monitoring of a single resolution of allylalanine monohydrate in ethanol/water (90 : 10). The dissolver was held at 20 °C and the crystallizer at 17 °C during crystallization and 23 °C during dissolution. Open circles denote the (*S*)-enantiomer (desired) and filled circles the (*R*)-enantiomer (undesired). The trace passes through a racemic baseline, single-enantiomer growth after (*S*)-seeding, a failure marked by the rise of the (*R*) signal from solid-phase (*R*)-allylalanine, and recovery on warming. Offline values of 84 : 16 and 94 : 6 e.r. confirm the online assignments.

Following acquisition of the second sample, 100 mg of enantiopure (*S*)-allylalanine seed crystals were introduced into the crystallizer. Immediately, the (*S*) signal increased while the (*R*) response remained constant at the saturation concentration. This behaviour is consistent with selective growth of the seeded (*S*) crystalline phase without formation of the counter enantiomer and demonstrates direct measurement of solid phase accumulation during the resolution.

After 1072 min, the (*R*) signal increased above the saturation baseline, indicating the appearance of (*R*)-allylalanine within the solid phase. As the dissolved (*R*)-allylalanine concentration remains constrained by the solubility equilibrium, this increase can only arise from solid-phase (*R*)-allylalanine captured during sampling. Operation was halted immediately following detection, and isolated solids were analysed offline, confirming an enantiomeric ratio (e.r.) of 84 : 16, in agreement with the DILC indication of contamination by the undesired enantiomer.

To restore enantiomeric purity, the crystallizer temperature was increased to 23 °C, promoting dissolution of the newly nucleated crystals. During this period both chromatographic signals decreased, while the (*R*) response returned to the saturation baseline, consistent with complete dissolution of the suspended (*R*) phase. Following this dissolution step, the recovered solid exhibited an improved enantiomeric ratio of 94 : 6.††The departure from enantiopure solid reflects insufficient deliquoring of the wet cake, which retains a thin film of racemic mother liquor. For a racemic conglomerate this residual film should be tolerated rather than washed away, because excessive washing of the solids selectively strips that racemic film and can erroneously inflate the reported enantiomeric ratio. Throughout the experiment, the DILC continuously quantified both the suspended crystalline phase and the mother liquor directly from a dense slurry. This capability provides information that is inaccessible using bulk optical methods such as polarimetry or circular dichroism, which measure only the net optical response of the bulk medium and are inherently influenced by temperature, solvent composition, and other optically active species rather than independently resolving the dissolved and solid phase compositions.

### Failure detection and recovery using a feedback control algorithm

With the diagnostic value of DILC established, we implemented a closed-loop controller that converts the real-time chromatographic readout into autonomous corrective action, removing the need for human supervision. The control problem is single-input, single-output: the manipulated input is the crystallizer temperature set point, which takes one of two discrete values, and the controlled signal is the chromatographic peak area of the undesired (*R*)-enantiomer sampled from the crystallizer, which serves as a real-time proxy for the solid phase enantiopurity. Because the saturation signal of the undesired enantiomer is nominally constant at fixed temperature, any sustained rise above the saturation baseline reports the onset of unwanted crystallization of the un-seeded enantiomer.

We therefore implemented a relay control strategy using a set threshold value (Fig. 6). At the start of each run, five baseline samples are acquired at the crystallization temperature define the in-control distribution of the (*R*)-signal. The failure threshold is set at the baseline mean plus four standard deviations (*T* = *µ* + 4*σ*). While the (*R*)-area remains below *T*, the system holds the crystallizer at the crystallization set point (17 °C) and samples at a long interval to maximize productivity. When the (*R*)-area crosses *T*, the controller switches the crystallizer to the dissolution set point (23 °C) and increases the sampling frequency, selectively redissolving the accreting undesired solid until the (*R*)-signal returns below threshold. The reactor is then returned to the crystallization set point and the DILC sampling frequency is reduced to maintain passive monitoring. Full implementation details and parameters are provided in the SI.

**Fig. 6:**
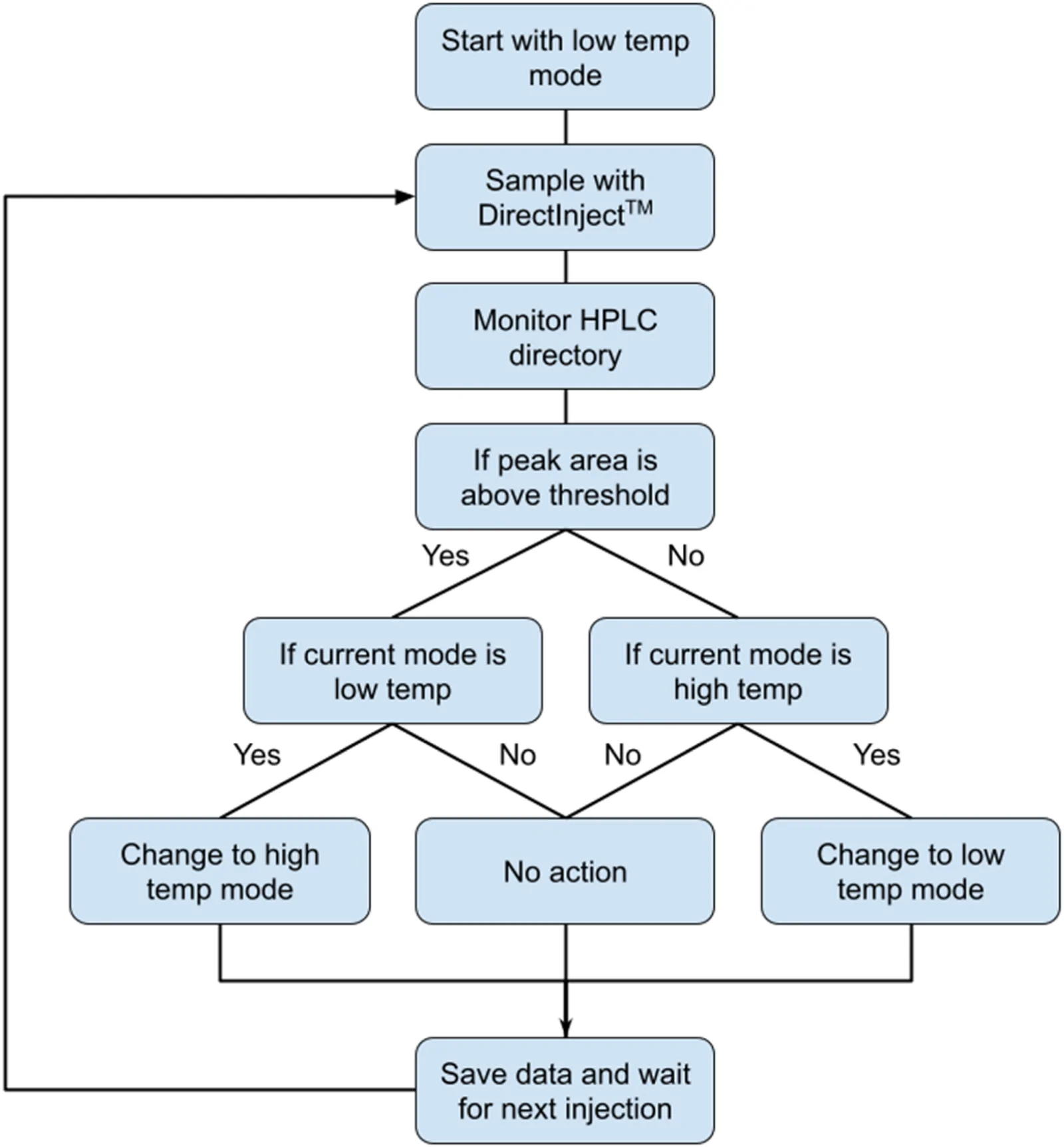
Decision logic of the autonomous CPC feedback controller. Each real-time DILC measurement of the undesired (*R*)-enantiomer peak area is compared against a data-derived threshold (*T* = *µ* + 4*σ*); crossing the threshold switches the crystallizer from the crystallization set point (17 °C) to the dissolution set point (23 °C), with an asymmetric sampling cadence in each state.

The analytical duty cycle sets the time resolution of this loop. The chiral separation is isocratic, so no gradient re-equilibration is required and the cycle time is governed by elution rather than column reconditioning. Both enantiomers elute within the 4.0 to 5.8 min integration window used by the controller, and the complete sample-to-result cycle, comprising withdrawal through the sampling probe, in-line dissolution, delivery, injection and integration, supports a minimum sampling interval of 10 min. The schedule is deliberately asymmetric. During recovery the system samples at this 10 min floor, whereas while the process is in control the interval was relaxed, initially to 1 h and subsequently to 2 h, to limit the analytical and solvent burden of a five-day campaign. Detection latency is therefore bounded by the in-control sampling interval. An event that begins and completes between injections is not time-resolved, but the counter-enantiomer it produces does not redissolve spontaneously at the crystallization set point, so the resulting solid-phase burden persists and is detected at the next injection. The consequence of discrete sampling is a bounded delay before correction rather than a failure to detect, and the interval may be shortened to the 10 min floor where faster response is required.

### Long-duration CPC performance and implications for scale-up

To test the platform under realistic operating conditions, we performed a multi-day continuous preferential crystallization of allylalanine monohydrate under DILC-enabled feedback control. The experiment ran for nearly five days, with the only major interruption arising from depletion of the nitrogen supply required for ELSD operation rather than from failure of the crystallization platform or control logic (Fig. 7). Over the multi-day campaign, the DILC record provided a continuous operating history of the resolution inside the crystallizer. Productive CPC was identified by progressive growth of the seeded enantiomer signal while the undesired enantiomer remained near its saturation baseline. In contrast, a sustained rise in the undesired-enantiomer signal indicated that counter-enantiomer crystals were beginning to accumulate in the sampled slurry. This event marked the onset of a recoverable process excursion and triggered a temperature-driven dissolution cycle. Recovery was confirmed when the undesired-enantiomer signal returned to its baseline value, after which the system resumed low-temperature crystallization. In this way, the chromatographic trace reports not only material accumulation, but also the controller's detection and correction of crystallization failure in real time.

**Fig. 7:**
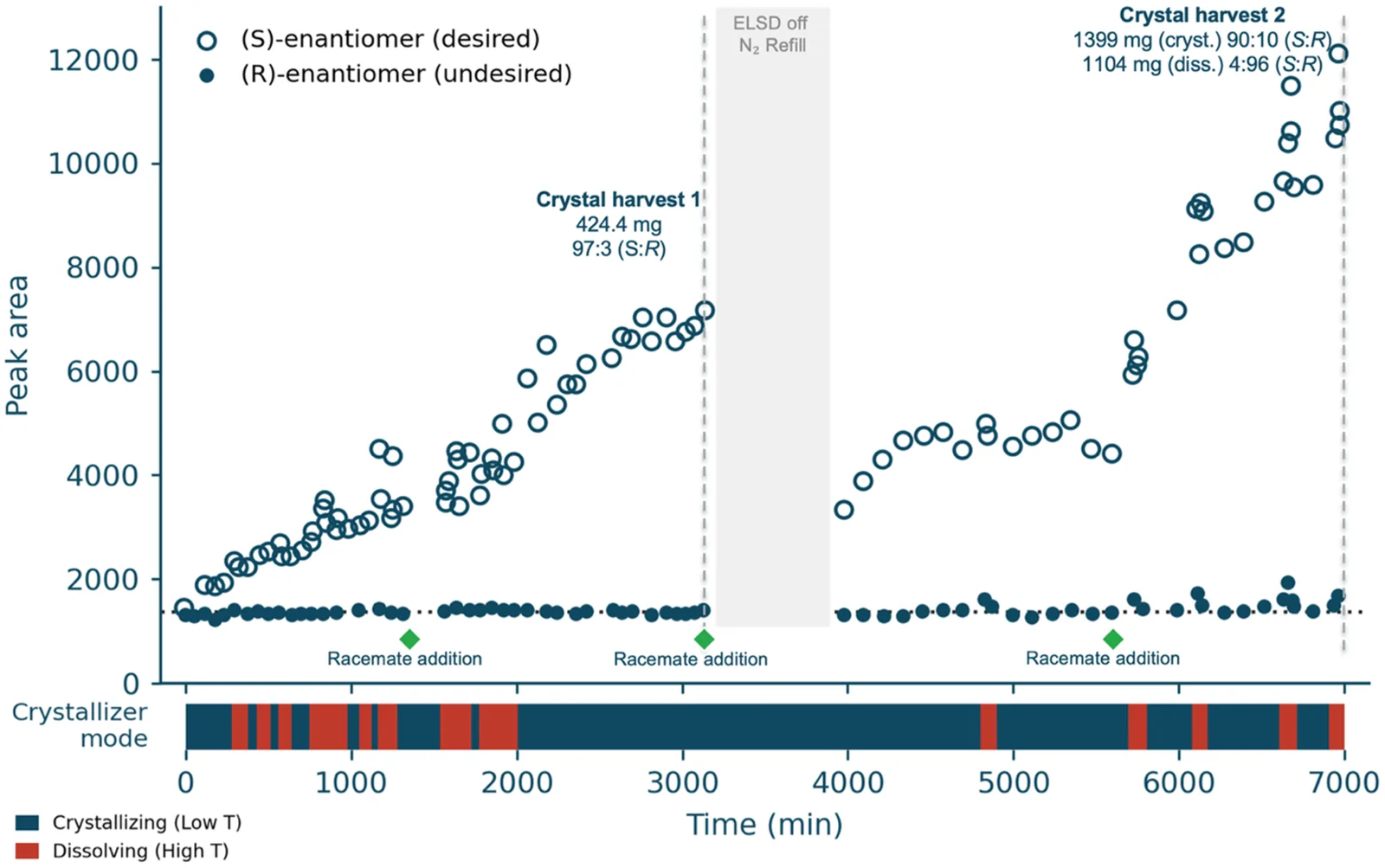
Multi-day CPC of allylalanine monohydrate monitored from the crystallizer by DILC. Open circles denote the seeded, desired enantiomer, and filled circles denote the undesired counter-enantiomer. The lower mode bar indicates controller state: low-temperature crystallization in blue and high-temperature dissolution/recovery in orange. The data gap from 3136 to 3976 min resulted from depletion of the ELSD nitrogen supply. Harvest annotations report isolated mass and enantiomeric ratio.

When the undesired-enantiomer signal rose above the defined threshold, the controller initiated a temperature-driven dissolution cycle. Recovery was confirmed when this signal returned to its saturation baseline, after which the crystallizer resumed low-temperature operation and productive growth of the seeded phase continued. This feedback loop converted spontaneous appearance of the counter-enantiomer from a potentially terminal failure mode into a detected and recoverable process excursion. The early portion of the campaign also showed that autonomous CPC requires a carefully defined operating envelope. During the first 1500 min, the controller transitioned frequently between crystallization and dissolution, which we attribute to two operational factors. The failure threshold was initially positioned too close to the saturation signal of the undesired enantiomer, increasing sensitivity to normal analytical variation. In addition, the temperature ramp to return from dissolution to crystallization was sufficiently aggressive that it could promote undesired nucleation during recovery. Refining these parameters produced clearer failure-and-recovery cycles in the later phase of the run, especially from 4000 to 7000 min.

Because allylalanine monohydrate can access more than one solid form, we also considered whether the repeated thermal cycling might drive a solution-mediated phase transformation. Phase identity was established by powder X-ray diffraction, and the superimposable patterns of the commercial (*S*)-enantiomer and the racemate confirm conglomerate behaviour. More directly, every racemate charge introduced during the campaign was verified by PXRD as the desired conglomerate monohydrate before addition, so phase identity was confirmed repeatedly across the five days rather than only at the outset. The thermal excursion is in any case a modest one, spanning 17 to 23 °C, with both vessels remaining in the region where the monohydrate is the stable form for the solvent composition used and with the crystallizer continuously seeded with the desired phase. The cycling therefore modulates supersaturation rather than carrying the system across a phase boundary, and no chromatographic signature of a solution-mediated transformation was observed at any point in the run.

The same DILC record also provided practical information needed to operate the process over extended time. Racemate replenishment was treated as a controlled unit operation rather than as an uncontrolled feed event. An initial *in situ* addition showed that feed preparation and equilibration could perturb the crystallizer if the dissolver had not fully returned to the intended state. Subsequent additions were therefore conducted using *ex situ* recrystallized material with PXRD confirmation before charging. This operational adjustment simplified material replenishment and reduced the likelihood that feed addition would introduce an uncontrolled solid form or concentration excursion into the autonomous CPC.

A second important feature of the long-duration trace is that the DILC signal reports not only failure, but also process completion under a given material charge. Growth of the desired-enantiomer signal plateaued at approximately 3000 and 5000 min, indicating that productive crystallization had slowed because the accessible desired enantiomer had been depleted from the circulating system. At this stage, continued operation at the crystallization set point no longer improves recovery and may increase the risk of undesired nucleation. The plateau therefore provides a direct analytical trigger for intervention, either through racemate addition, harvest, or a change in operating state. In future implementations, this feature could be incorporated into the control algorithm so that plateau detection automatically prompts feed replenishment, harvest scheduling, or movement of the system away from supersaturation.

Two solid harvests were conducted during the campaign. The first harvest, taken from the crystallizer, provided 424.4 mg of the desired enantiomer at 97 : 3 e.r. A second harvest from both vessels provided gram-scale quantities of both enantiomers, with 1399 mg isolated from the crystallizer at 90 : 10 (*S* : *R*) and 1104 mg isolated from the dissolver at 4 : 96 (*S* : *R*). These results demonstrate that the platform can sustain productive operation over multiple days and provide access to both enantiomers in highly enriched form with limited user intervention.

Assessing yield in a preferential crystallization requires care, because isolated mass measured against total charge understates the performance of the process. The system operates at saturation throughout, so the working volume retains a fixed inventory of dissolved material that cannot be recovered by filtration at any point. Using the measured solubilities of 9.56 mg mL^−1^ at the 17 °C crystallization set point and 10.21 mg mL^−1^ at 20 °C, together with the 120 mL working volume, this saturated inventory amounts to 1.24 g. Only material present in excess of the solubility limit is available for isolation. On that basis, of the 5.10 g of racemate charged, 3.86 g was isolable and 2.92 g was isolated, a recovery of 76%. Because the solution in equilibrium with a conglomerate is racemic, the dissolved inventory is divided equally between the two enantiomers, and the per-enantiomer recoveries are 1.82 g of the 2.28 g available for (*S*), or 80%, and 1.10 g of the 1.58 g available for (*R*), or 70%. Referenced to the 120 mL working volume over the five-day campaign, the space-time yield is approximately 4.9 g L^−1^ d^−1^ of isolated solid, of which approximately 3.0 g L^−1^ d^−1^ is the desired (*S*)-enantiomer.

The platform does not change the thermodynamics of the resolution. The solubility limit, the saturated inventory and the attainable enantiopurity are fixed by the phase behaviour. What feedback control adds is the ability to keep approaching that ceiling across a multi-day campaign rather than surrendering it at the first failure.

The harvest data also served as an endpoint diagnostic. The crystallizer harvest was of lower enantiomeric ratio than the dissolver harvest, indicating that the undesired enantiomer was not present solely as a separate crystalline phase removable by thermal recovery. Progressive dissolution of the second crystallizer harvest gave a persistent undesired-enantiomer signal until the solid had completely dissolved, consistent with partial incorporation into the lattice of the desired phase.^21^ Such solid-solution behaviour places a ceiling on attainable enantiopurity that no control strategy can lift. The platform nonetheless sustained the resolution for several days and delivered gram-scale quantities of both enantiomers, with the controller responding to the rising undesired-enantiomer burden as it would to any other excursion.

Overall, the long-duration experiment demonstrates that DILC-enabled CPC can function as an adaptive resolution platform rather than a fixed crystallization recipe. The online chromatographic signal reports productive growth, detects the onset of counter-enantiomer crystallization, verifies recovery after thermal dissolution, identifies when the available charge has been depleted, and reveals non-ideal solid-phase behaviour that directly affects process design. These capabilities are central to scale-up, where extended operation increases the likelihood of nucleation events, feed perturbations, and phase-behaviour complications. By combining real-time chiral analysis with a recoverable crystallizer-dissolver architecture, this platform provides a practical route toward autonomous, self-correcting preferential crystallization of systems that would otherwise require continual expert oversight.

## Conclusions

In this work, we establish an autonomous platform for continuous preferential crystallization by combining direct-inject liquid chromatography with feedback-controlled crystallization and dissolution. The central advance is that DILC provides a real-time, enantioselective readout of the resolution as it proceeds. By sampling directly from the slurry and analyzing the material by chiral LC, the system reports both the progress of the crystallization and the evolving enantiomeric composition of the crystalline phase. This allows the platform to identify when productive growth has plateaued, when material should be harvested or replenished, and when the undesired enantiomer has begun to accumulate. Such information is exceptionally difficult to obtain in automated crystallization workflows, since solid-phase enantiopurity normally requires manual isolation, drying, and offline analysis. Here, DILC converts that traditionally offline measurement into an *operando* signal that can be used directly for process monitoring and control.

Coupling this measurement to a crystallizer and dissolver architecture enabled autonomous execution of a complex chiral resolution that would normally require continual expert oversight. The system did not simply follow a fixed temperature program. Instead, it operated as a goal-directed process in which the desired state and recovery conditions were defined in advance. When the chromatographic signal for the undesired enantiomer remained below threshold, the platform continued crystallization and promoted growth of the seeded major enantiomer. When that signal rose above threshold, the controller initiated a temperature-driven dissolution cycle to remove newly formed undesired solid before returning the system to productive crystallization. In this way, spontaneous nucleation of the counter-enantiomer was converted from a terminal failure into a detectable and recoverable process excursion. Applied to allylalanine monohydrate, this strategy enabled multi-day operation with minimal intervention and produced gram-scale quantities of highly enantioenriched material.

A particularly significant outcome was the ability of the feedback-controlled CPC platform to navigate a system showing partial solid-solution behaviour. In a classical preferential crystallization, incorporation of even small amounts of the undesired enantiomer into the growing crystal phase can compromise the resolution, since the process no longer behaves as an ideal conglomerate and solid purity cannot be reliably inferred from endpoint analysis alone. In this study, real-time DILC revealed this non-ideal behaviour during operation and allowed the controller to continue exploiting the kinetic advantage of accretion onto the seeded major enantiomer. When growth of the undesired enantiomer rose above the defined threshold, a thermal recovery cycle was triggered to dissolve newly accumulated undesired material and restore productive operation. This enabled the system to progressively advance an unusually challenging resolution that would otherwise require expert judgement and manual intervention.

Taken together, these results show that online chiral chromatography can function as more than an analytical endpoint assay. When integrated with feedback control, it becomes a decision-making signal for autonomous chiral crystallization. The combination of high-information DILC analysis, a physically recoverable CPC architecture, and threshold-based thermal control provides a strategy for self-correcting resolution processes in which both progress and failure can be detected directly from the operating slurry. More broadly, this work demonstrates how structure-specific online analytics can move autonomous chemical processes beyond fixed recipes and toward adaptive operation, where complex crystallizations are monitored, corrected, and advanced with a level of responsiveness normally requiring expert human oversight.

## Author contributions

J. S. Derasp – conceptualization, methodology, investigation, writing – original draft. F. Painsecq – investigation, software. W. Zhang – investigation. M. E. Reish – software, methodology. P. Prieto – investigation. J. E. Hein – conceptualization, supervision, funding acquisition, writing – review & editing.

## Conflicts of interest

Prof. Jason Hein is also the founder and CTO of Telescope Innovations, a chemical technology company.

## Supplementary Material

SC-OLF-D6SC05647D-s001

SC-OLF-D6SC05647D-s002

## Data Availability

CCDC 2572940 and 2572941 contain the supplementary crystallographic data for this paper.^36*a*,*b*^ The data supporting this article have been included as part of the supplementary information (SI). Supplementary information is available. See DOI: https://doi.org/10.1039/d6sc05647d.
