# Supplementary material for: Direct-inject liquid-chromatography enables an autonomous continuous preferential crystallization platform
Source: Chem Sci. 2026 Sep 28. Online ahead of print. doi: 10.1039/d6sc05647d (PMC13647125; doi:10.1039/d6sc05647d)
Supplement: Supplementary file 1 [file SC-OLF-D6SC05647D-s001.pdf]

Supplementary Information (SI) for Chemical Science.
This journal is © The Royal Society of Chemistry 2026
Supplementary Information (SI) for Chemical Science.
This journal is © The Royal Society of Chemistry

# Supporting Information

## Direct-Inject Liquid-Chromatography Enables an Autonomous Continuous Preferential Crystallization Platform

Joshua S. Derasp, Felix Painsecq, Ivory Zhang, Matt Reish, Paloma Prieto and Jason E. Hein *
*Department of Chemistry, University of British Columbia, Vancouver, BC, Canada.*

### Table of contents

S1. General Remarks
S2. Allylalanine Synthesis and Derivatization
S3. CPC Platform
S4. Solubility Data for rac-α-allylalanine monohydrate
S5. Procedure to Probe EasySampler Data Acquisition (Figure 4)
S6. CPC Procedure (Figure 5)
S7. Analysis of Harvested Material
S8. Progressive Dissolution Experiment
S9. Mass Spectrum Chromatogram
S10. PXRD: commercial (*S*)-(−)- vs rac-α-allylalanine
S11. LC-MS Chromatograms of N-Protected Allylalanine
S12. Single-Crystal Structures
S13. NMR Spectra
S14. References

### S1. General Remarks

**Chemical suppliers:** All reagents and solvents were purchased from Fisher Scientific, Alfa Aesar, Sigma-Aldrich, Combi-blocks, and VWR and used without further purification. Optima-grade water and methanol were used as HPLC solvents.

**NMR:** Spectra were recorded on a Bruker Avance 300 (300 MHz / 75 MHz for $^{1}$H / $^{13}$C) or Bruker Avance 400 (400 MHz for $^{1}$H), calibrated to residual solvent peaks. Coupling constants in Hz.

**PXRD:** Bruker D8-Advance diffractometer; Bragg-Brentano; Cu $K\alpha_1/K\alpha_2$; Ni filter; LynxEye Si-strip detector; 40 kV, 40 mA; 0.6 mm divergence / 8 mm anti-scatter slits, 2.5° Soller; basic stage.

**Equipment:** Mettler-Toledo EasyMax 102 glass reactors (50/100/250 mL) with Teflon covers, submersible thermocouple, magnetic stir bar, controlled by iControl 6.1. Automated sampling: DirectInject-LC (Telescope Innovations) with an Agilent 1290 Infinity HPLC and Agilent 385-ELSD detector.

**HPLC methods:** (i) Astec CHIROBIOTIC T, 15 cm × 4.6 mm, 5 µm; 25 °C; A = water, B = methanol; 1.0 mL/min; isocratic 10% A / 90% B. (ii) CHIRALCEL OJ-RH, 15 cm × 4.6 mm, 5 µm; 25 °C; A = water + 0.1% AcOH, B = MeCN + 0.1% AcOH; 0.5 mL/min; isocratic 60% A / 40% B. ELSD (all methods): evaporator 50 °C, nebulizer 50 °C, gas 1.6 SLM, data rate 80 Hz, LED 100%, smoothing 50 (5.0 s), PMT gain 1.0.

## S2. Allylalanine Synthesis and Derivatization

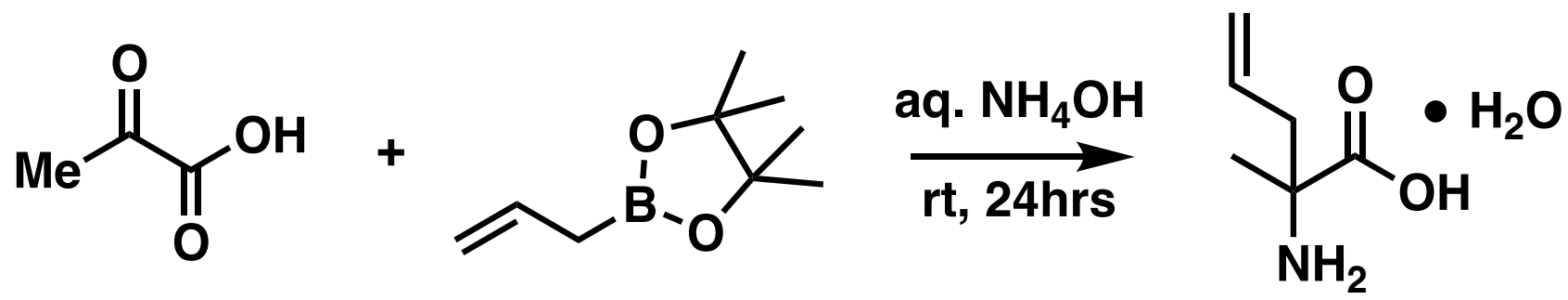


**Scheme S1.** Petasis synthesis of (rac)-α-allylalanine monohydrate.

**Synthesis of *rac*-α-allylalanine, *rac*-4.**

To a mixture of 2-oxopropanoic acid (46.41 mmol, 4.087 g) in 28% ammonium hydroxide solution (90 mL) was added 2-allyl-4,4,5,5-tetramethyl-1,3,2-dioxaborolane (69.61 mmol, 11.70 g, 1.5 eq). The reaction medium was stirred at room temperature for 24 h. After completion of the reaction, the reaction medium was evaporated under reduced pressure. Three cycles of addition of ethanol (3 × 30 mL) and evaporation were then carried out. Purification of the crude product was achieved by recrystallization from a 2-propanol/water mixture (90:10). Filtration afforded 1.62 g of colourless crystals, a 27% yield.

$^1$H NMR (400 MHz): δ = 5.81–5.59 (m, 1H), 5.32–5.14 (m, 2H), 2.70–2.32 (dd, 2H), 1.45 (s, 3H) ppm.

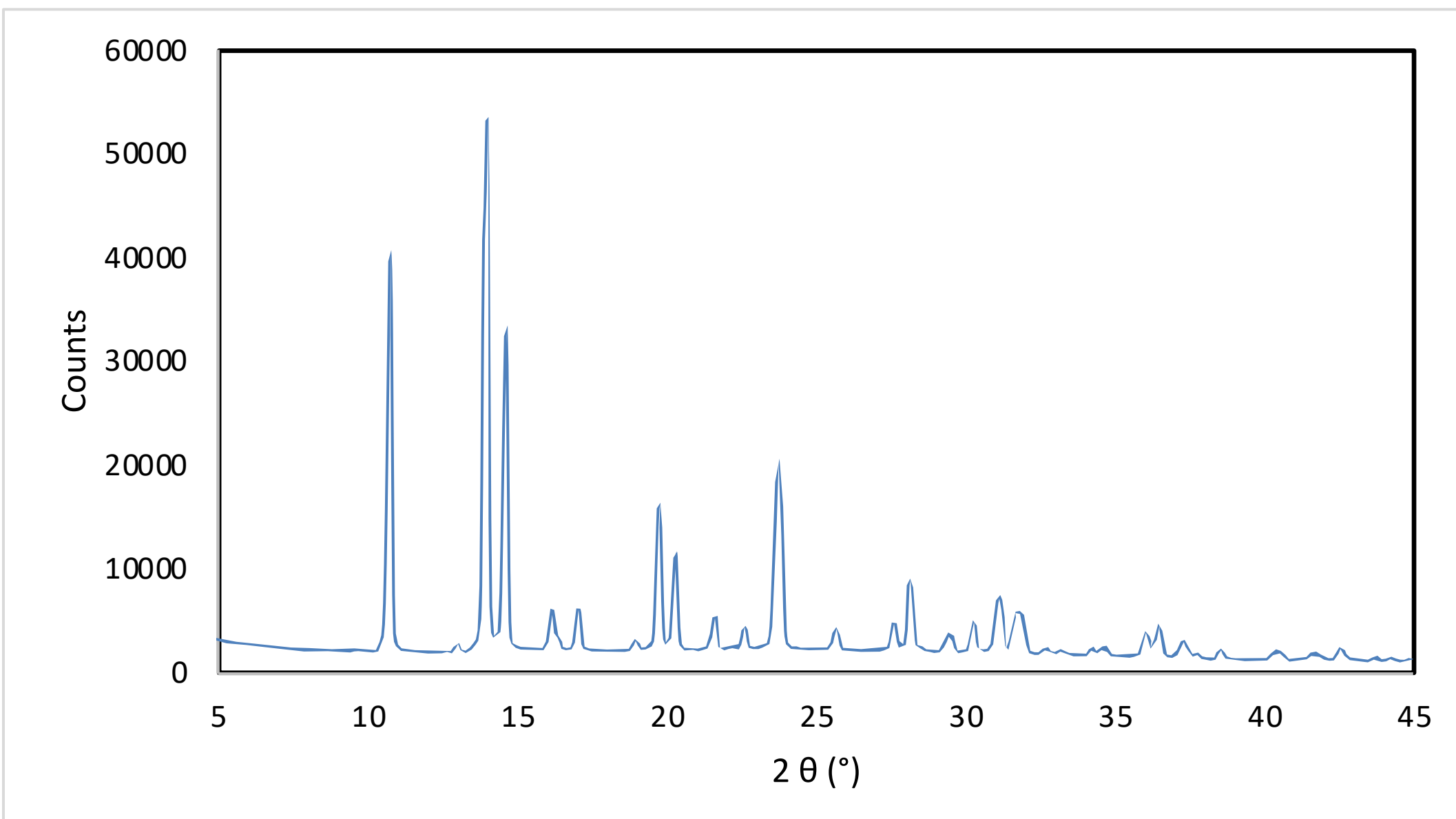


**Figure S1.** PXRD of recrystallized rac-α-allylalanine monohydrate.

## S3. CPC Platform

**Platform.** From left to right: SF-10 Vapourtec pumps; two-position four-port valves; EasyMax 102 (Mettler Toledo); EasySampler probe (Mettler Toledo); DirectInject (Telescope Innovations); HPLC (Agilent 1290 Infinity); ELSD (Agilent 385). Two EasyMax reactors give high-level temperature control (left = dissolver, right = crystallizer). SF-10 pumps plumb into the two-position four-port valves connecting both reactors; valves on a timer switch flow direction. UHMW-PE 10 µm filters at each tubing line ensure transfer of solid-free supernatant. The EasySampler probe samples the crystallizer; a variable-interval sampling algorithm communicates with the DILC parent code to obtain and deliver samples to the HPLC.

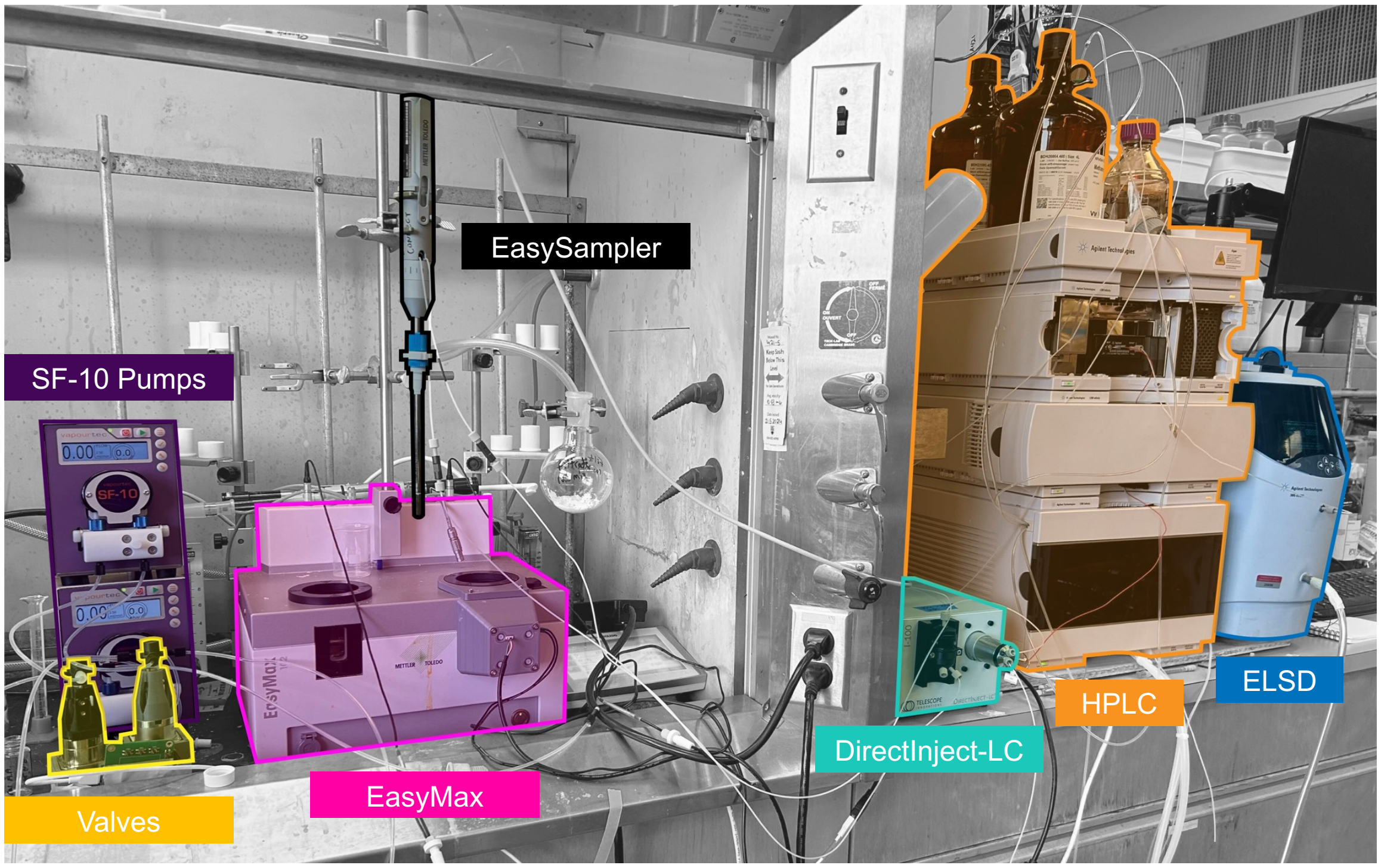


**Figure S2.** Annotated photograph of the CPC platform.

**Flow reversal across the transfer filters.** The reciprocating-flow valve arrangement used to keep the transfer filters clear is described in the main text (Figure 4). Operating parameters were as follows: filters, UHMW-PE, 10 µm; valves, four-way PEEK bulkhead diagonal-flow (IDEX V-101D), one per transfer line; toggle interval, 15 min, applied to both valves simultaneously. The peristaltic pumps run continuously at constant rate and direction throughout, so only the tubing segment between each valve and its vessel changes flow direction.

**Valve actuation.** Two geared stepper motors (42BYGH40-18-22A-C5.18, 5.18:1 gear ratio) are coupled to the valve stems through a 3D-printed adapter and a machined shaft coupler. Homing and feedback control use AS5600 magnetic encoders held in a 3D-printed housing that also retains the motors, with a radial magnet bonded to the rear of each motor shaft. Motor control and encoder readout use an in-house stepper-driver PCB, and the switching interval is set from a Python class. The valve assembly CAD (https://gitlab.com/heingroup/flow-reversal-valves), the PCB design files (https://gitlab.com/heingroup/stepper-driver/-/tree/main/Design%20Files) and the dual-valve firmware and control class (https://gitlab.com/heingroup/stepper-driver/-/tree/main/Dual%20Valve%20Firmware) are all available on the Hein group GitLab.

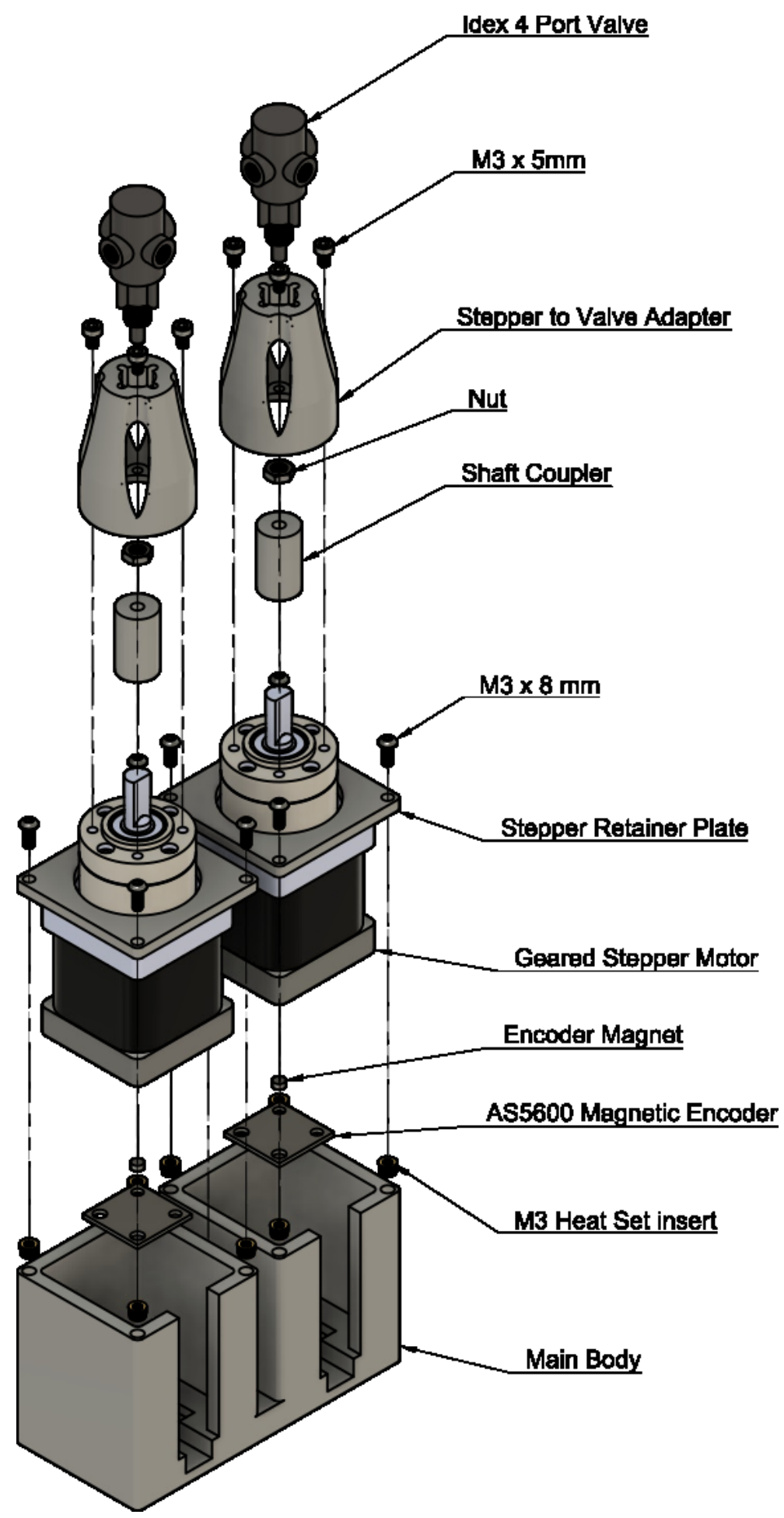


**Figure S3.** Exploded view of the custom two-valve actuator assembly.

**Bill of materials** for the flow-reversal valve assembly.

| Part | Qty | Part number | Source |
|---|---|---|---|
| 3D-printed PLA parts | 5 | — | https://gitlab.com/heingroup/flow-reversal-valves |
| Geared stepper motor | 2 | 42BYGH40-18-22A-C5.18 | RobotShop (DFRobot bipolar stepper with planetary gearbox) |
| Magnetic encoder and magnet | 2 | AS5600 | HiLetgo AS5600 magnetic encoder |
| 4-way PEEK bulkhead diagonal-flow valve | 2 | V-101D | IDEX Health & Science |
| M3 × 8 mm screw | 8 | 91292A112 | McMaster-Carr |
| M3 × 6 mm screw | 8 | 91292A111 | McMaster-Carr |
| M3 heat-set insert | 8 | 94180A331 | McMaster-Carr |

**Software requirements.** EasyMax controlled via the beta Reactor Device Server 1.0 (Mettler Toledo) over OPC-UA (requires EasyMax firmware 6.2), using the asyncua Python package; the Reactor Device Server must be running on a networked computer before starting. DirectInject controlled by a Telescope Innovations Python script sending HTTP requests to the DirectInject server.

**Workflow / parameters.** HPLC_PATH; THRESHOLD; LOW_TEMP (e.g. {“temp”: 17, “sample_per_n_min”: 120}); HIGH_TEMP; PEAK_RANGE (e.g. (4.0, 5.8)); DI_HOST; DELIVERY_VOLUME; LOOP_VOLUME. The system starts in low-temperature mode, initiates an injection, monitors HPLC output in the background, and extracts peak areas in the retention-time window. If a peak area exceeds the threshold in low-temp mode, it switches to high-temp mode (dissolution); if below threshold in high-temp mode, it returns to low-temp mode; otherwise no action.

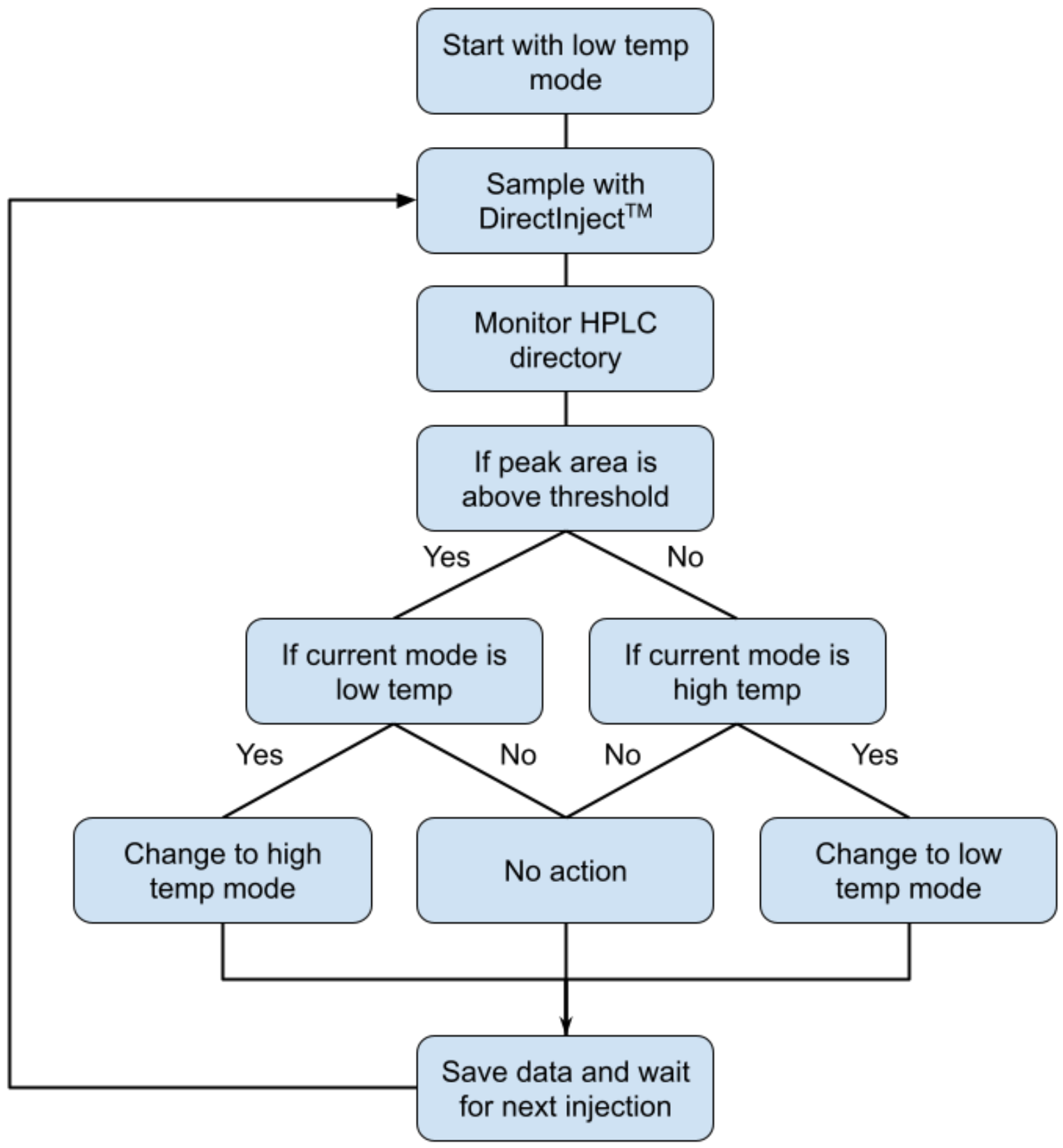


**Figure S4.** Automated CPC workflow.

## S4. Solubility Data for rac-α-allylalanine monohydrate

**Calibration curve.** 3.0 g substrate → 25 mL volumetric (60 mg/mL per enantiomer stock), diluted to 40, 20, 10, 5 mg/mL; each sampled 5× by DILC. ELSD response is exponential, so a log/log plot gave a linear area-vs-concentration correlation.

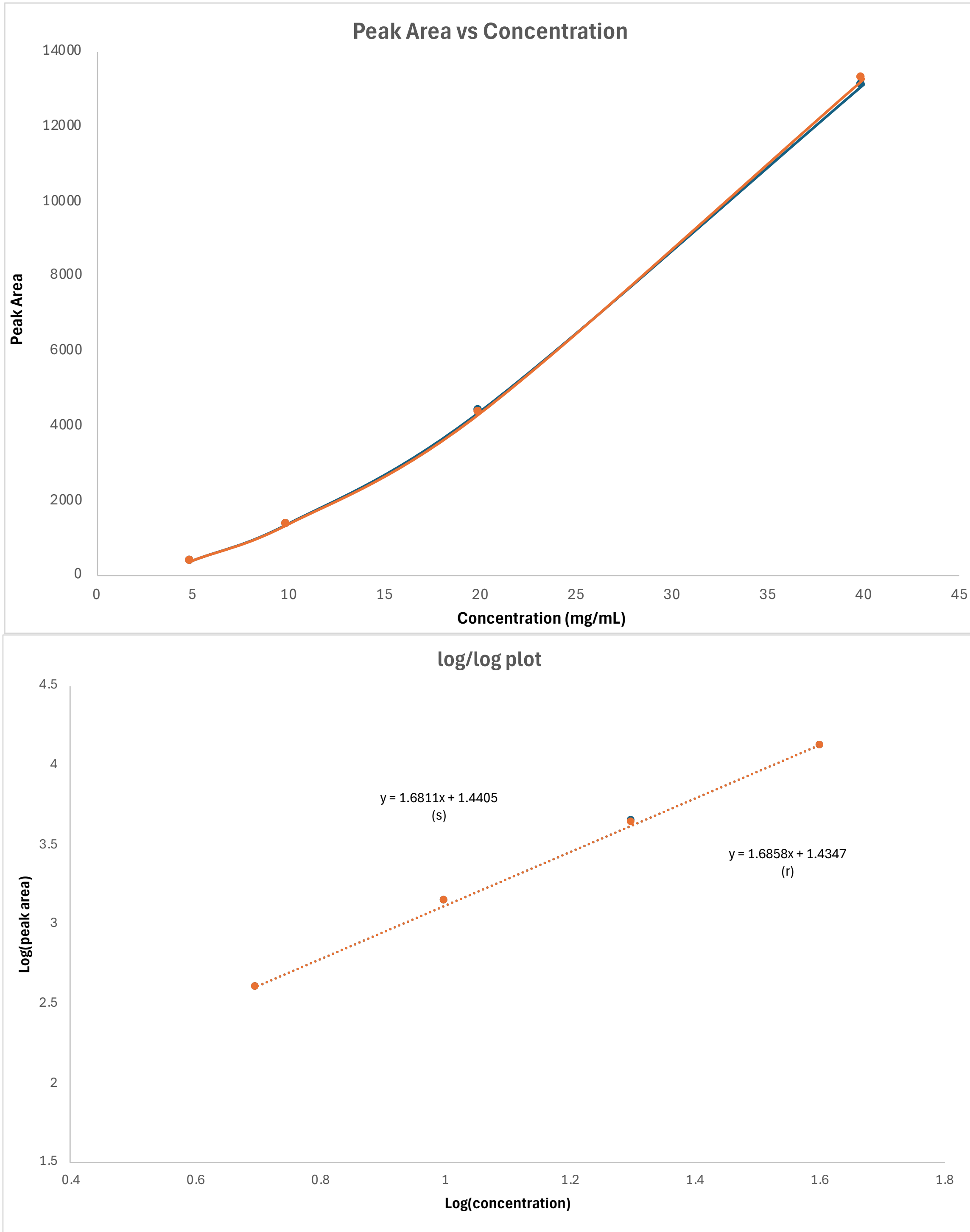


**Figure S5.** Peak area vs concentration (top) and log/log linearization (bottom) for rac-α-allylalanine monohydrate. Orange = (*R*), Blue = (*S*).

**Solubility curve.** 100 mL reactor, 3.0 g substrate + 75 mL 90:10 EtOH:water, 500 rpm; heated to 50 °C (20 min), cooled to 10 °C over 30 min, equilibrated 16 h. Solid-free supernatant pumped (SF-10, UHMW-PE 10 µm filter) to a second reactor, sampled in triplicate; returned, temperature raised in 3 °C increments (30 min equilibration) and repeated.

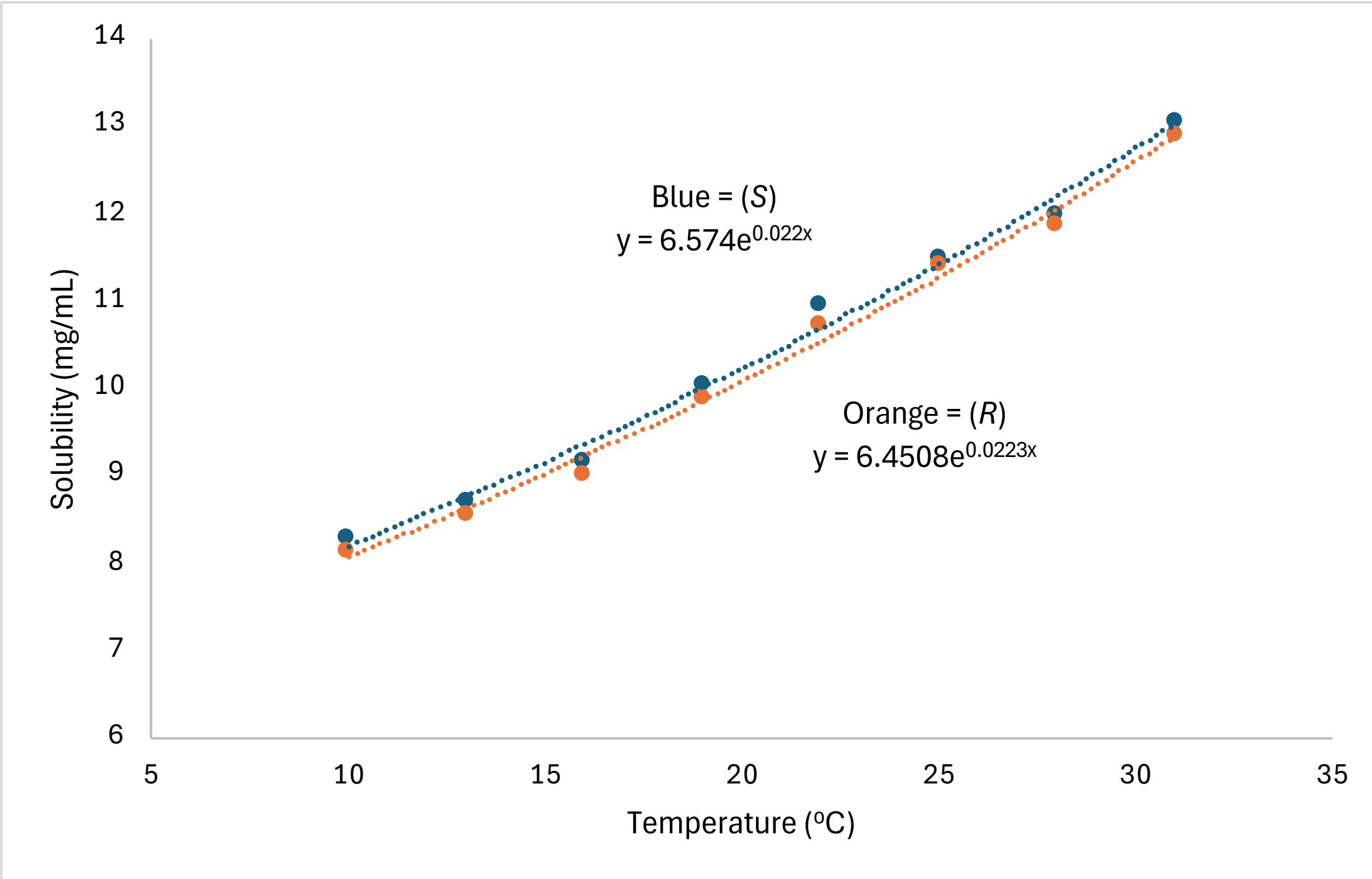


**Figure S6.** Solubility of rac-α-allylalanine vs temperature.

**Supersaturation temperature.** Same setup; supernatant pumped to a second reactor at 20 °C, then cooled 1 °C/10 min and monitored 1 h per step until crystallization. Primary nucleation occurred shortly after 16 °C. Set points: dissolver 20 °C (10.21 mg/mL), crystallizer 17 °C (9.56 mg/mL), dissolution 23 °C (10.90 mg/mL).

## S5. Procedure to Probe EasySampler Data Acquisition (Figure 4)

3.0 g rac-α-allylalanine monohydrate in a 100 mL EasyMax reactor; 120 mL 90:10 EtOH:water; sealed with a Teflon 5-port adapter; heated to 50 °C to full dissolution, cooled to 20 °C over 20 min, equilibrated overnight. Next day, solid-free supernatant pumped (2.5 mL/min) dissolver→crystallizer until half transferred, then a second SF-10 pump started (2.5 mL/min); crystallizer at 20 °C; 30 min equilibration. Crystallizer ramped 20→17 °C over 5 min; monitored 30 min for kinetic stability. Sampling via iCLC every 8 min (first 5 samples) then every 20 min. 100 mg enantiopure (*S*)-seed added between samples 2 and 3. On clear (*R*)-signal deviation, system paused (crystallizer → 21 °C, pumps stopped); 200 µL sampled into a filtered Eppendorf, centrifuged, solid dissolved in water and injected (HPLC). After confirming loss of solid-phase enantiopurity (84:16 e.r.), crystallizer raised to 23 °C and pumping/sampling resumed. On regaining saturation signal, paused again; manual sample showed restored enantiopurity (94:6 e.r.).

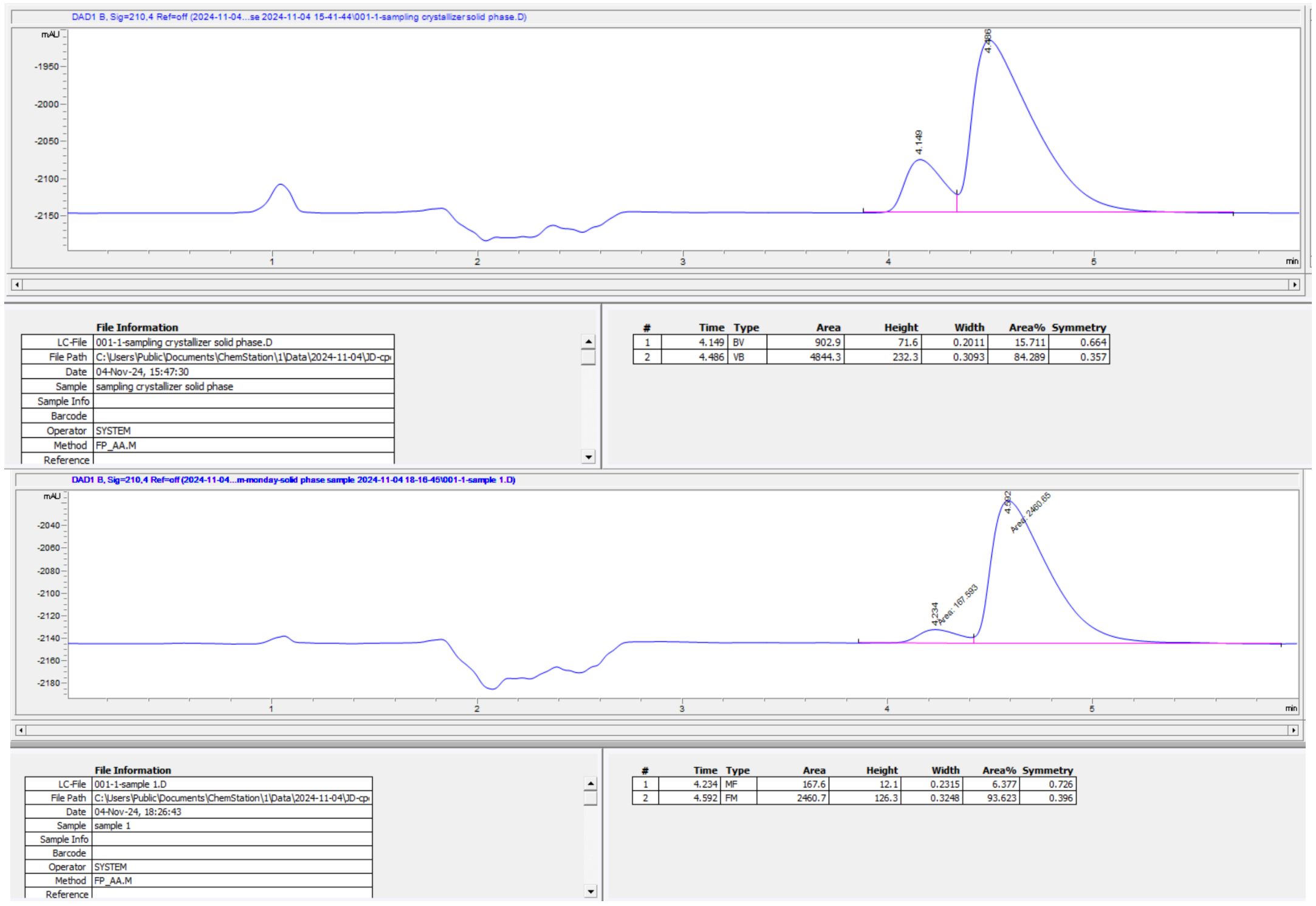


**Figure S7.** Manual samples showing loss (84:16 e.r., top) and restoration (94:6 e.r., bottom) of solid-phase enantiopurity.

## S6. CPC Procedure (Figure 5)

Setup as S5 (3.0 g substrate, 120 mL 90:10 EtOH:water, both reactors stirred 500 rpm). After establishing the crystallizer at 17 °C, ~45 min monitoring captured five baseline samples to set the threshold; the CPC control algorithm was started and 100 mg (*S*)-seed added. The run was largely autonomous except for three interventions: (1) periodic ELSD nitrogen-dewar depletion (data gaps); (2) racemate addition to the dissolver; (3) crystal harvests.

**Setting the threshold.** Threshold = average saturation signal + (4 × standard deviation) from 5 samples; system-specific, must be optimized per system.

**Dynamic sampling interval.** Dissolution phase fixed at 10 min (≈ fastest given the HPLC method length); crystallization phase 1 h initially, later 2 h to limit throughput impact.

**Racemate addition.** Initial in situ recrystallization proved too slow (>2 h equilibration needed) and was replaced by ex situ recrystallization with PXRD confirmation of the conglomerate phase before each addition.

**Solid-phase isolation.** Pumps stopped, crystallizer → 21 °C; one SF-10 transfers solution to the dissolver, leaving filtered solids in the crystallizer (a portion dried under air for mass; significant solid left behind for productivity). Dissolver solids isolated analogously.

## S7. Analysis of Harvested Material

**Throughput.**

| Input | Mass (g) | Output | Mass (g) |
|---|---|---|---|

| | | | |
|---|---|---|---|
| Initial loading | 3.0 + 0.10 seed | 1st harvest | 0.424 (*S*) |
| 1st addition | 1.0 | 2nd harvest | 1.40 (*S*) |
| 2nd addition | 1.0 | | 1.10 (*R*) |
| Total | 5.1 | | 2.92 |
| Total by enantiomer* | 2.9 (*S*) / 2.2 (*R*) | | 1.82 (*S*) / 1.1 (*R*) |

*Racemate was slightly (*S*)-enriched from prior experimentation (56% S).

**Enantiomeric ratio (HPLC):** harvest 1 (crystallizer) 97:3; harvest 2 (crystallizer) 90:10; harvest 2 (dissolver) 4:96 (*S*:*R*)

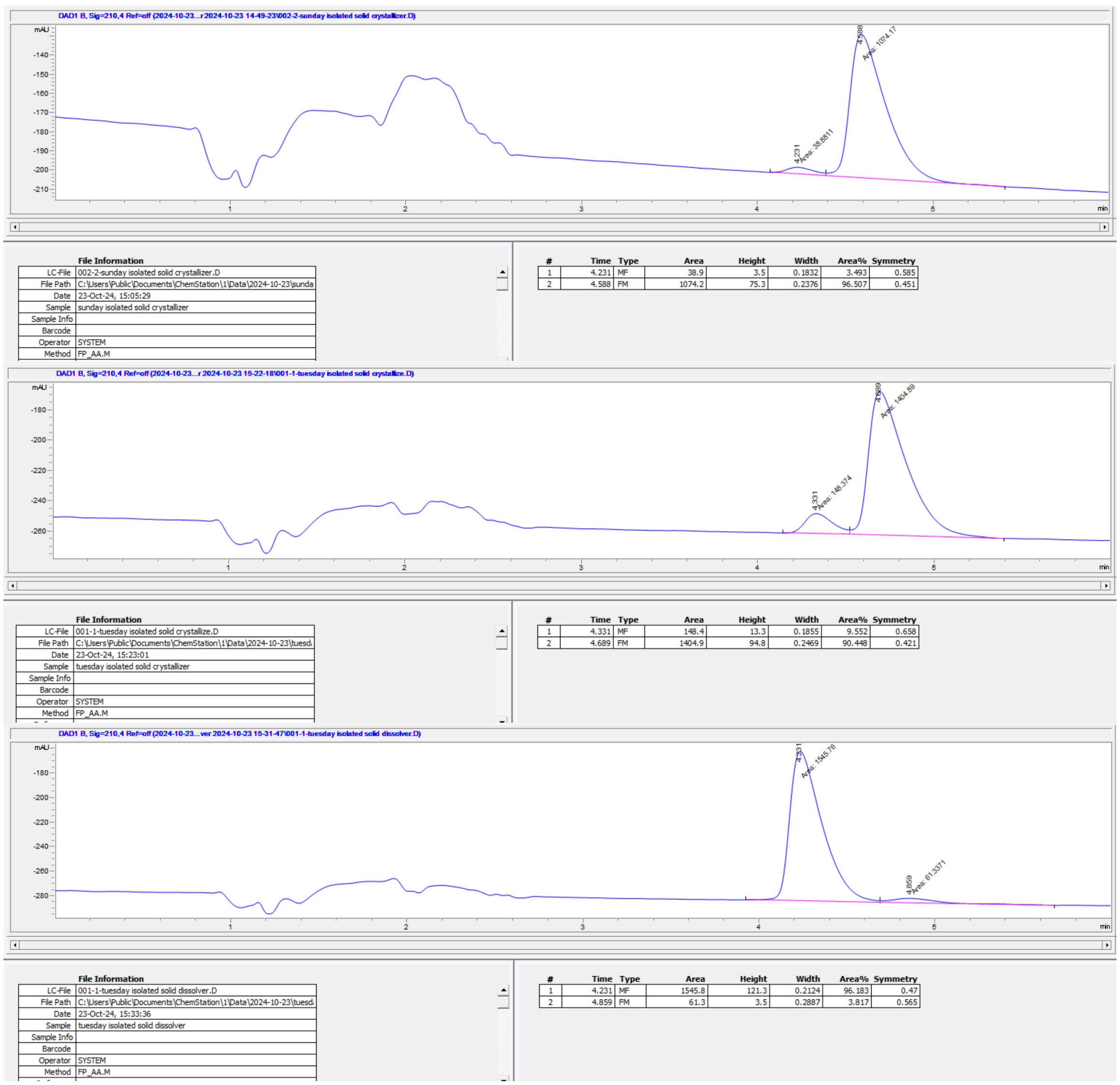


**Figure S8.** HPLC chiral HPLC analysis of harvested material (harvest 1 crystallizer; harvest 2 crystallizer; harvest 2 dissolver).

## S8. Progressive Dissolution Experiment

50 mg of second-crystallizer-harvest solid (90:10 e.r.) + stir bar in an Eppendorf; 500 µL 90:10 EtOH:water added, stirred 500 rpm 15 min, centrifuged, liquid decanted for HPLC; solid resuspended and the cycle

repeated to full dissolution (12 cycles). A persistent (*R*)-signal across cycles 3–12 indicates a solid solution (undesired enantiomer embedded in the (*S*) lattice), unresolvable by a dissolution campaign.

| Cycle | % Area (*S*) | % Area (*R*) |
|---|---|---|
| 1 | 56% | 44% |
| 2 | 83% | 17% |
| 3 | 97% | 3% |
| 4–7 | 98% | 2% |
| 8 | 99% | 1% |
| 9–12 | ~98% | ~2% |

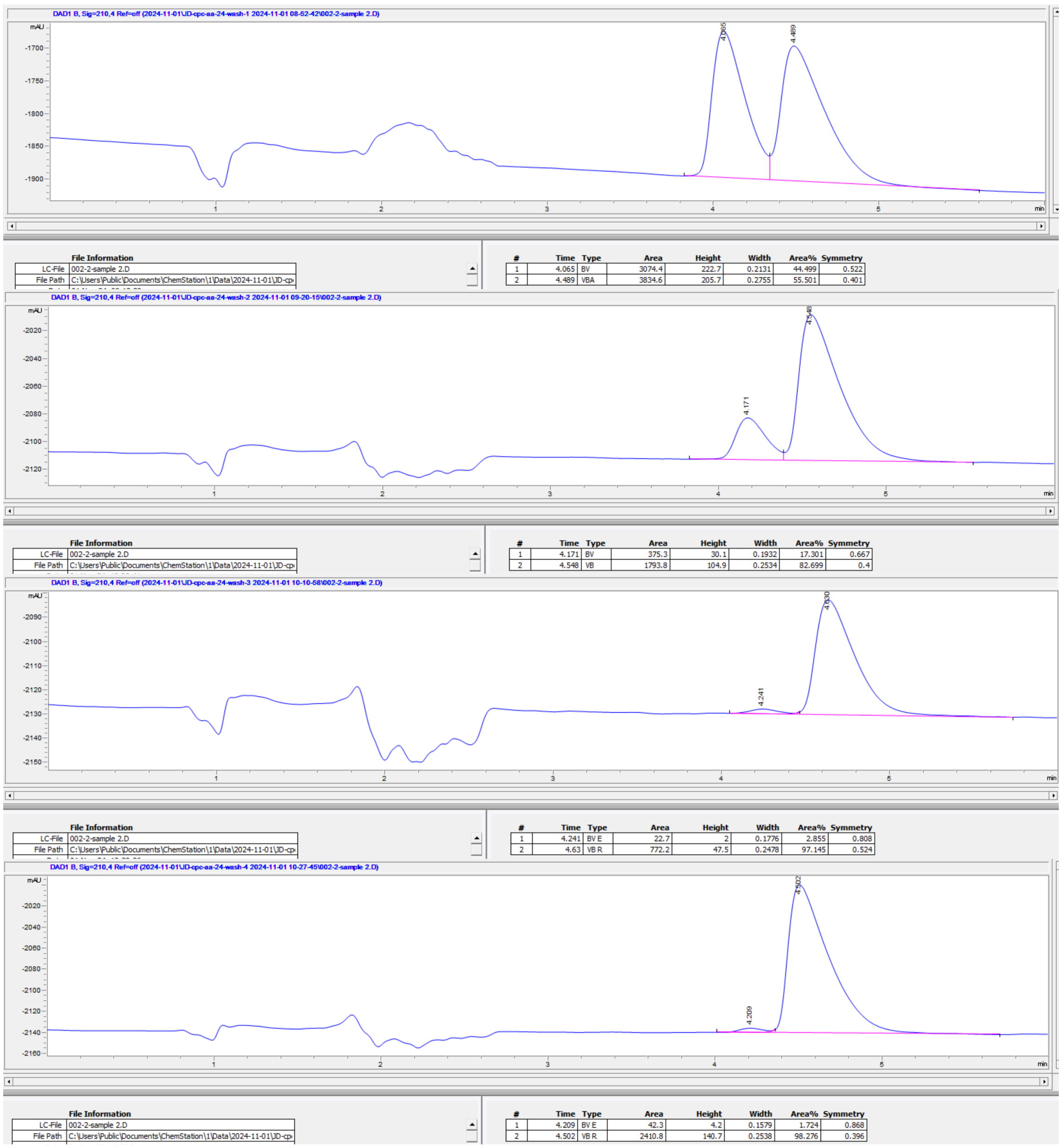

DAD1 B, Sig=210,4 Ref=off (2024-11-01\JD-cpc-aa-24-wash-5 2024-11-01 10-52-05\002-2-sample 2.D)

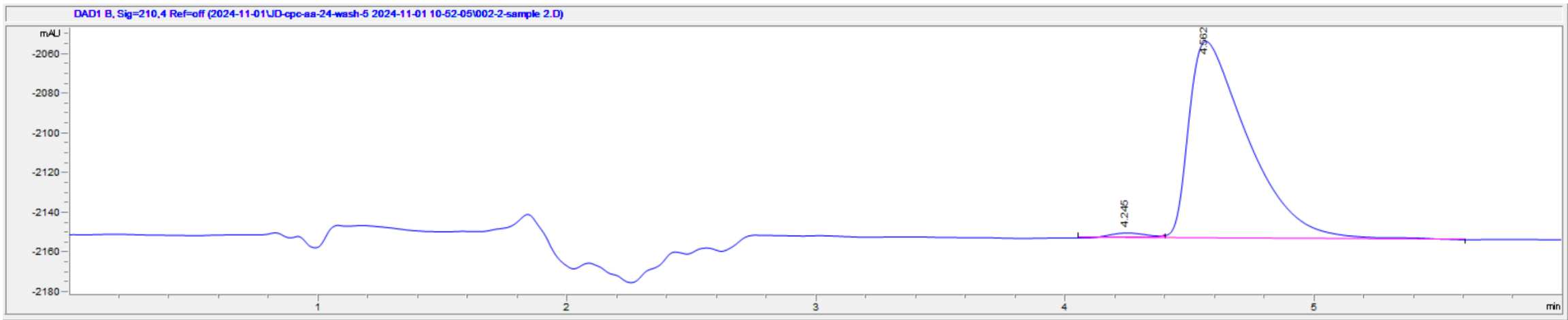


| File Information | |
|---|---|
| LC-File | 002-2-sample 2.D |
| File Path | C:\Users\Public\Documents\ChemStation\1\Data\2024-11-01\JD-cp |

| # | Time | Type | Area | Height | Width | Area% | Symmetry |
|---|---|---|---|---|---|---|---|
| 1 | 4.245 | BV E | 28.7 | 2.7 | 0.1699 | 1.702 | 0.849 |
| 2 | 4.562 | VB R | 1657.2 | 99.8 | 0.2457 | 98.298 | 0.424 |

DAD1 B, Sig=210,4 Ref=off (2024-11-01\JD-cpc-aa-24-wash-6 2024-11-01 12-55-44\002-2-sample 2.D)

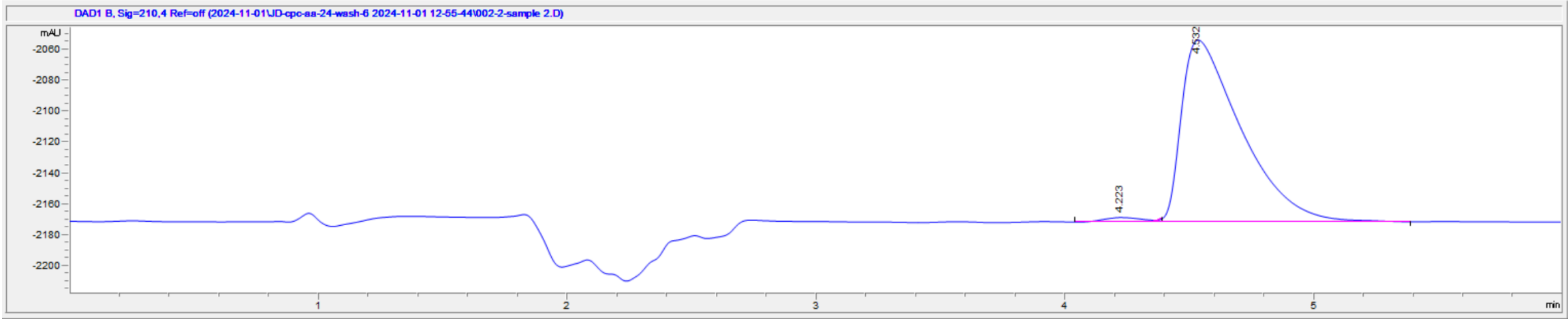


| File Information | |
|---|---|
| LC-File | 002-2-sample 2.D |
| File Path | C:\Users\Public\Documents\ChemStation\1\Data\2024-11-01\JD-cp |

| # | Time | Type | Area | Height | Width | Area% | Symmetry |
|---|---|---|---|---|---|---|---|
| 1 | 4.223 | BV E | 32.1 | 3 | 0.1682 | 1.605 | 0.832 |
| 2 | 4.532 | VB R | 1968.8 | 118 | 0.2466 | 98.395 | 0.409 |

DAD1 B, Sig=210,4 Ref=off (2024-11-01\JD-cpc-aa-24-wash-7 2024-11-01 13-16-37\002-2-sample 2.D)

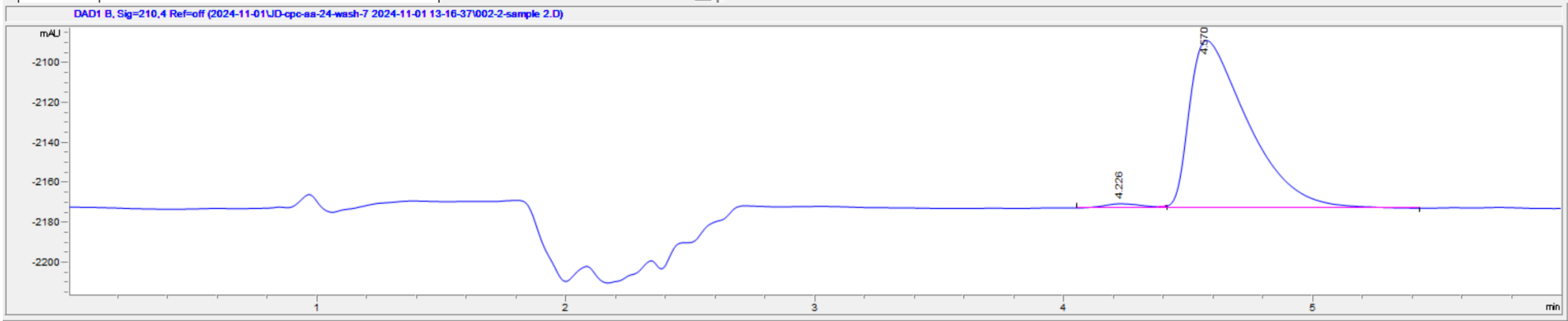


| File Information | |
|---|---|
| LC-File | 002-2-sample 2.D |
| File Path | C:\Users\Public\Documents\ChemStation\1\Data\2024-11-01\JD-cp |

| # | Time | Type | Area | Height | Width | Area% | Symmetry |
|---|---|---|---|---|---|---|---|
| 1 | 4.226 | BV E | 22.7 | 2.1 | 0.1708 | 1.620 | 0.753 |
| 2 | 4.57 | VB R | 1376.9 | 84 | 0.2433 | 98.380 | 0.435 |

DAD1 B, Sig=210,4 Ref=off (2024-11-01\JD-cpc-aa-24-wash-8 2024-11-01 14-10-19\002-2-sample 2.D)

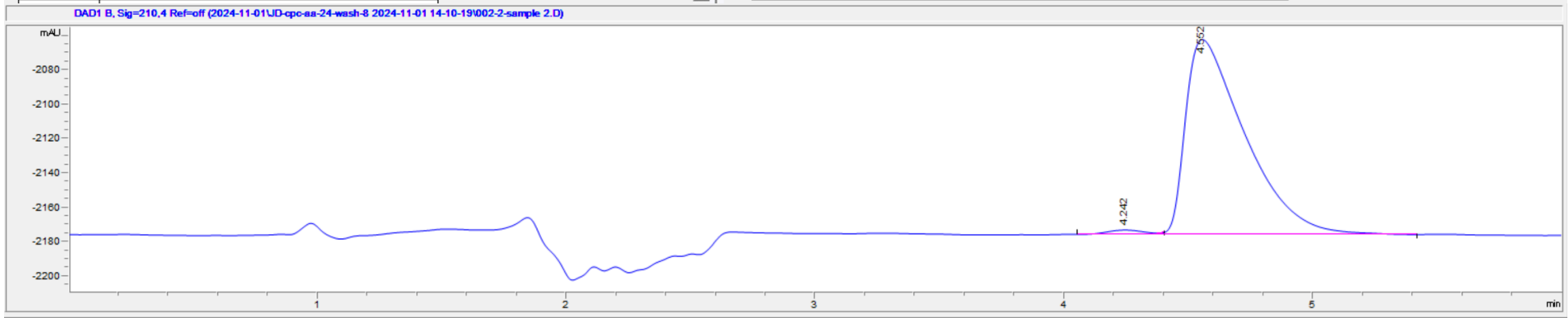


| File Information | |
|---|---|
| LC-File | 002-2-sample 2.D |
| File Path | C:\Users\Public\Documents\ChemStation\1\Data\2024-11-01\JD-cp |

| # | Time | Type | Area | Height | Width | Area% | Symmetry |
|---|---|---|---|---|---|---|---|
| 1 | 4.242 | BV E | 25.2 | 2.5 | 0.1581 | 1.326 | 0.937 |
| 2 | 4.552 | VB R | 1878.9 | 113.5 | 0.2471 | 98.674 | 0.405 |

DAD1 B, Sig=210,4 Ref=off (2024-11-01\JD-cpc-aa-24-wash-9 2024-11-01 15-42-09\001-1-sample 1.D)

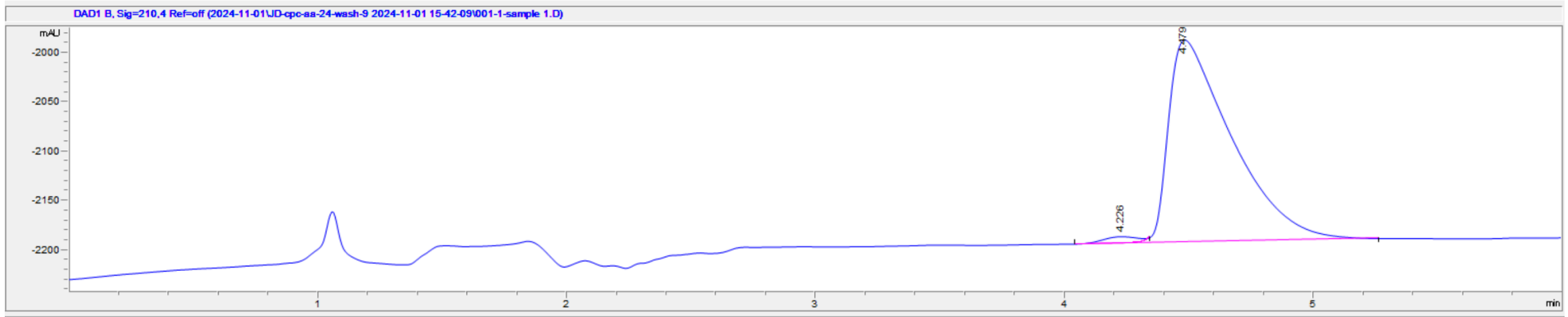


| File Information | |
|---|---|
| LC-File | 001-1-sample 1.D |
| File Path | C:\Users\Public\Documents\ChemStation\1\Data\2024-11-01\JD-cp |

| # | Time | Type | Area | Height | Width | Area% | Symmetry |
|---|---|---|---|---|---|---|---|
| 1 | 4.226 | BV E | 64.1 | 6.6 | 0.1553 | 1.743 | 1.13 |
| 2 | 4.479 | VB R | 3612.6 | 205 | 0.2535 | 98.257 | 0.368 |

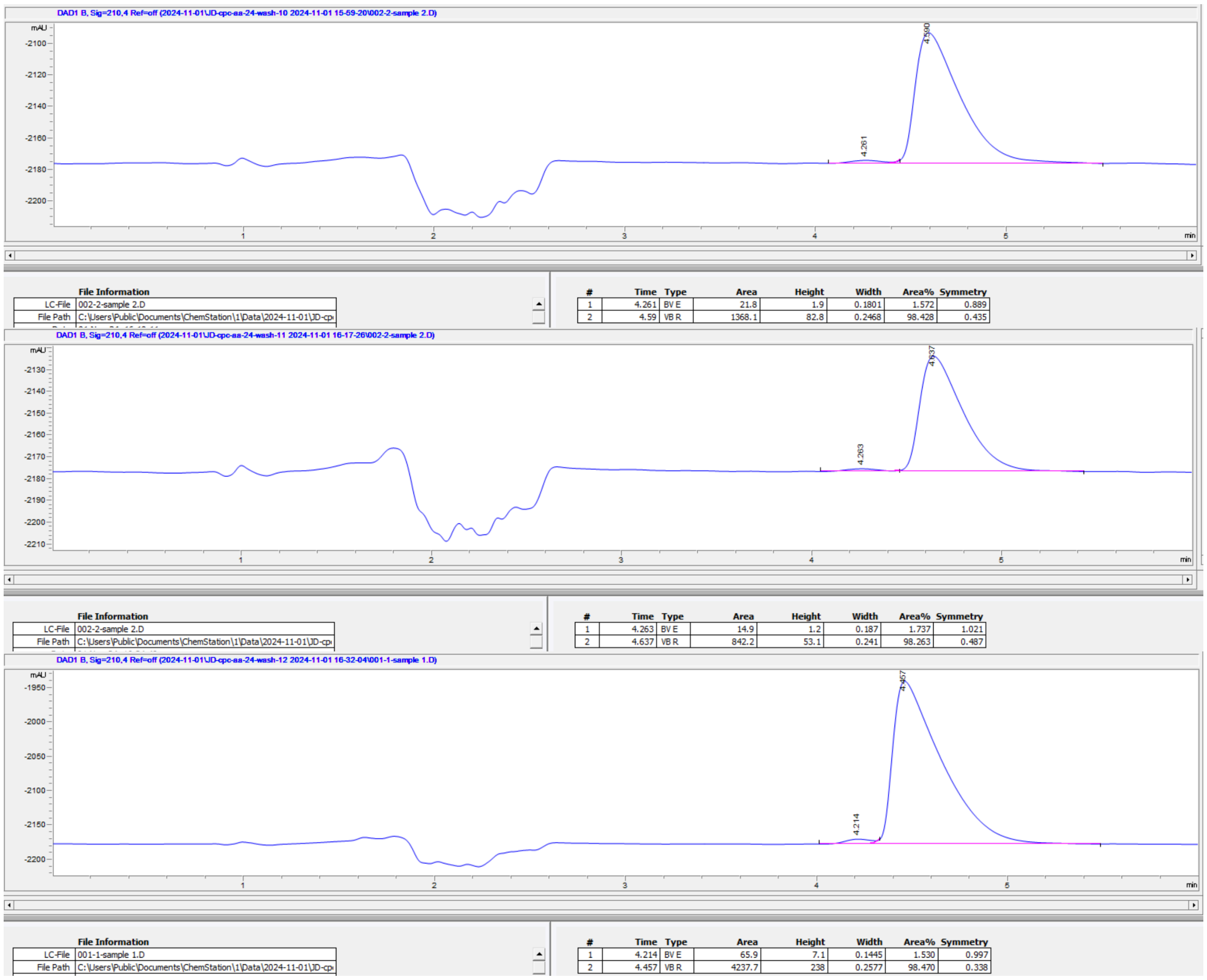


**Figure S9.** HPLC traces, progressive dissolution cycles 1–12.

## S9. Mass Spectrum Chromatogram

As a secondary validation MS chromatograms of rac-α-allylalanine were recorded on a LCMS system using the Astec CHIROBIOTIC T column at four process points: crystallizer start, dissolver start, crystallizer end, dissolver end.

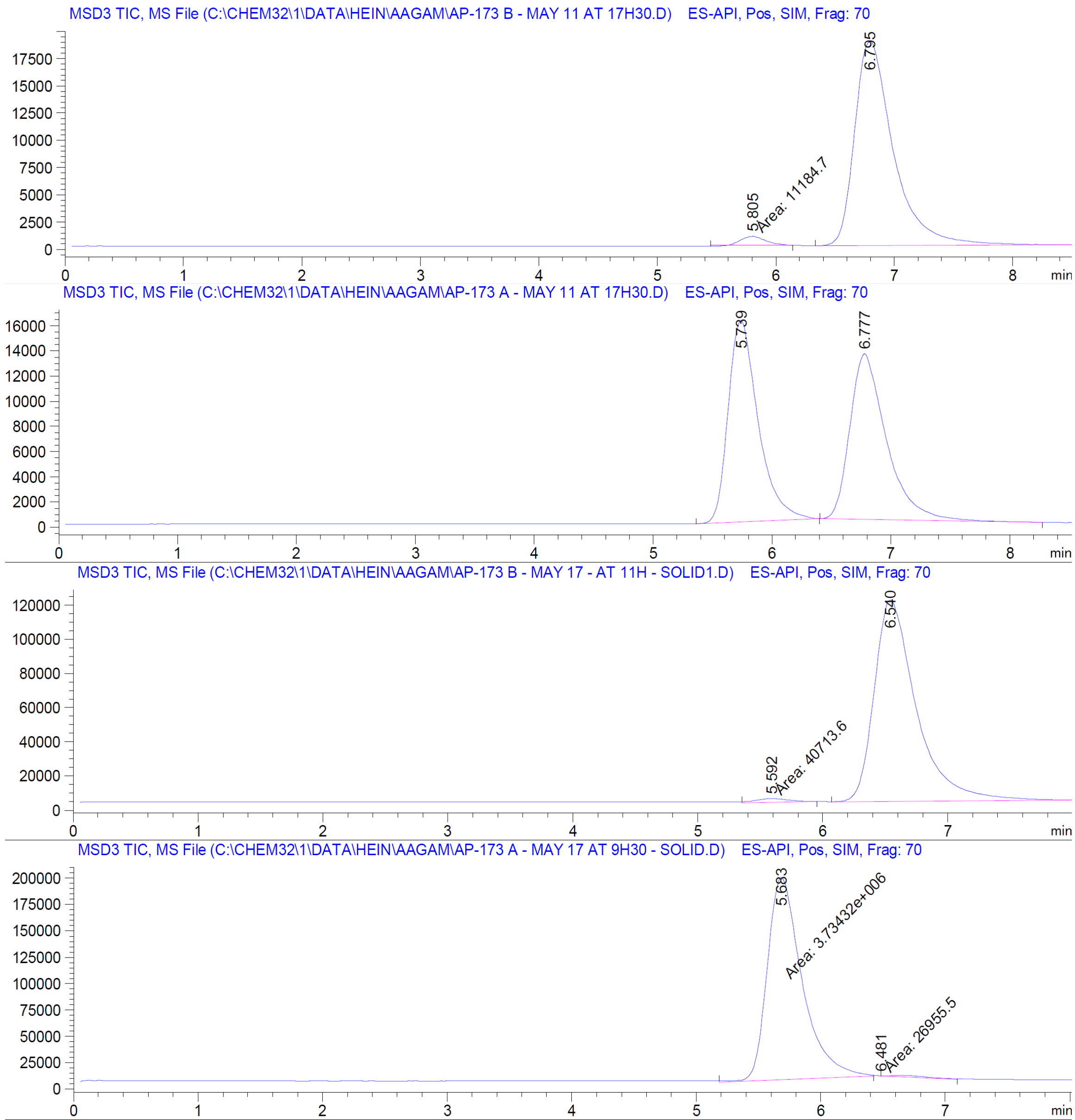


**Figure S10.** MS chromatograms at four CPC process points.

## S10. PXRD: commercial (*S*)-(−)- vs rac-α-allylalanine

The XRPD pattern for the enantiopure amino acid was generated from its crystal structure (Mercury); the rac pattern was measured on the Bruker D8-Advance. The patterns were superimposable, confirming rac-α-allylalanine is a conglomerate.

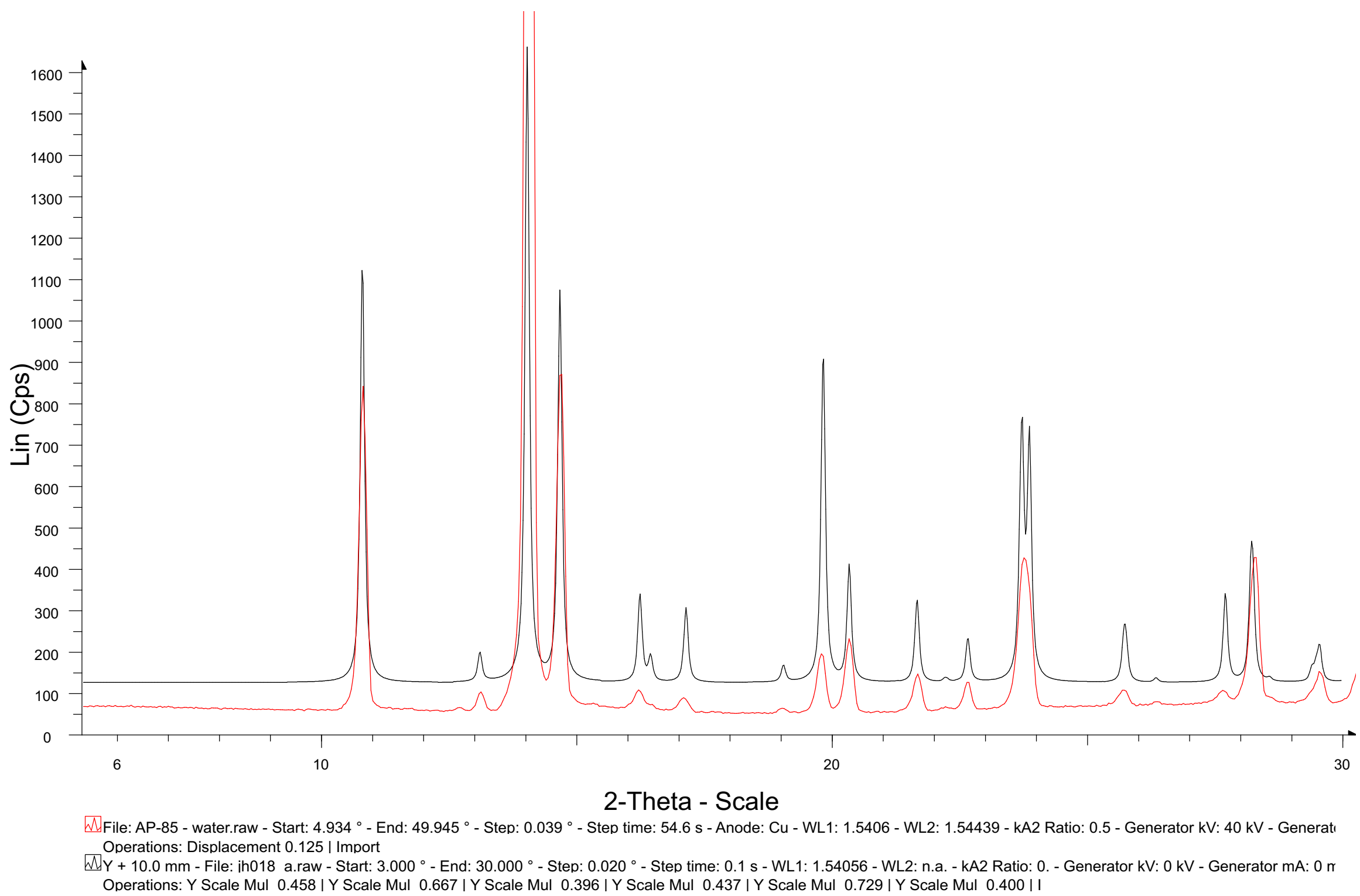


**Figure S11.** Overlaid PXRD patterns of commercial (*S*)-(−)- (RED) and rac-α-allylalanine (BLACK).

## S11. LC-MS Chromatograms of N-Protected Allylalanine

As an alternative method for e.e. determination LC-MS of Cbz-protected α-allylalanine on the CHIRALCEL OJ-RH column can be used. MS-based monitoring needs no derivatization but suffers ion-suppression quantification issues; Cbz derivatization gave quantitative determination of the enantiomeric ratio. Traces: Cbz-(*R*)-, Cbz-(*S*)-, co-injection at differing concentrations, and benzyl alcohol.

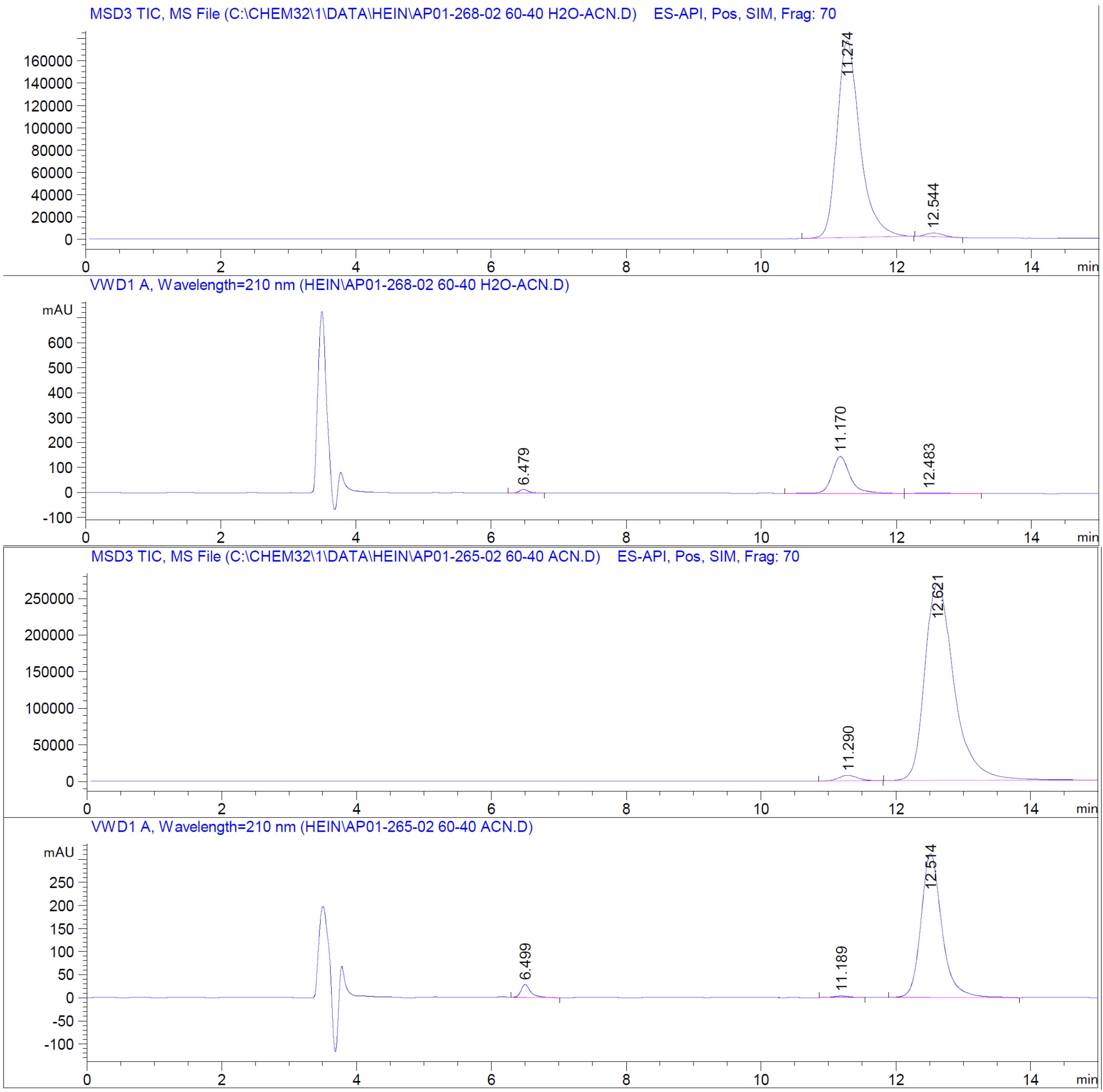
MSD3 TIC, MS File (C:\CHEM32\1\DATA\HEIN\AP01-268-02 60-40 H2O-ACN.D) ES-API, Pos, SIM, Frag: 70
11.274
12.544
VWD1 A, Wavelength=210 nm (HEIN\AP01-268-02 60-40 H2O-ACN.D)
mAU
6.479
11.170
12.483
MSD3 TIC, MS File (C:\CHEM32\1\DATA\HEIN\AP01-265-02 60-40 ACN.D) ES-API, Pos, SIM, Frag: 70
11.290
12.621
VWD1 A, Wavelength=210 nm (HEIN\AP01-265-02 60-40 ACN.D)
mAU
6.499
11.189
12.514
min

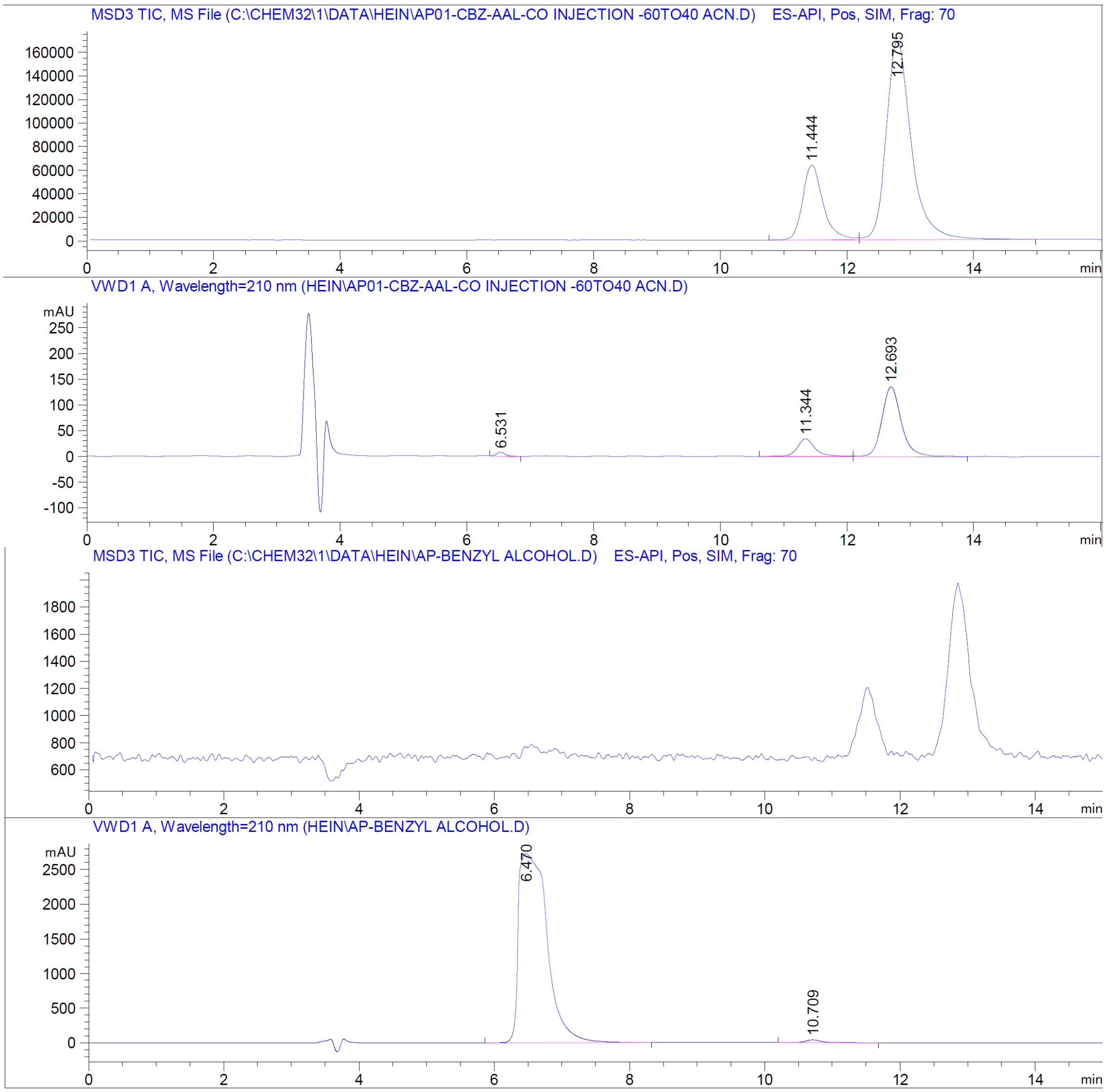


**Figure S12.** LC-MS of Cbz-protected allylalanine enantiomers and benzyl alcohol.

## S12. Single-Crystal Structures

### Crystal data — cu_jh008_0m_a (from rac-α-allylalanine, 4, CCDC 2572940).

| Parameter | Value |
|---|---|
| Formula / Mass | $C_6H_{13}NO_3$ / 147.17 |
| Crystal system | Tetragonal |
| a, b, c /Å | 8.9191(4), 8.9191(4), 20.7003(10) |
| Volume /Å³ | 1646.72(17) |
| Space group | $P4_32_12$ |
| Z | 8 |
| $R_1$ (I>2σ) / wR(F²) | 0.0483 / 0.1379 |
| GooF / Flack | 1.083 / 0.65(10) |

**Crystal data — jh018_a (from (*S*)-α-allylalanine; CCDC 2572941).**

| Parameter | Value |
|---|---|
| Formula / Mass | $C_6H_{13}NO_3$ / 147.17 |
| Crystal system | Tetragonal |
| a, b, c /Å | 8.92490(10), 8.92490(10), 20.6780(3) |
| Volume /Å³ | 1647.08(4) |
| Space group | $P4_12_12$ |
| Z | 8 |
| $R_1$ (I>2σ) / wR($F^2$) | 0.0668 / 0.1922 |
| GooF / Flack | 1.097 / 0.21(12) |

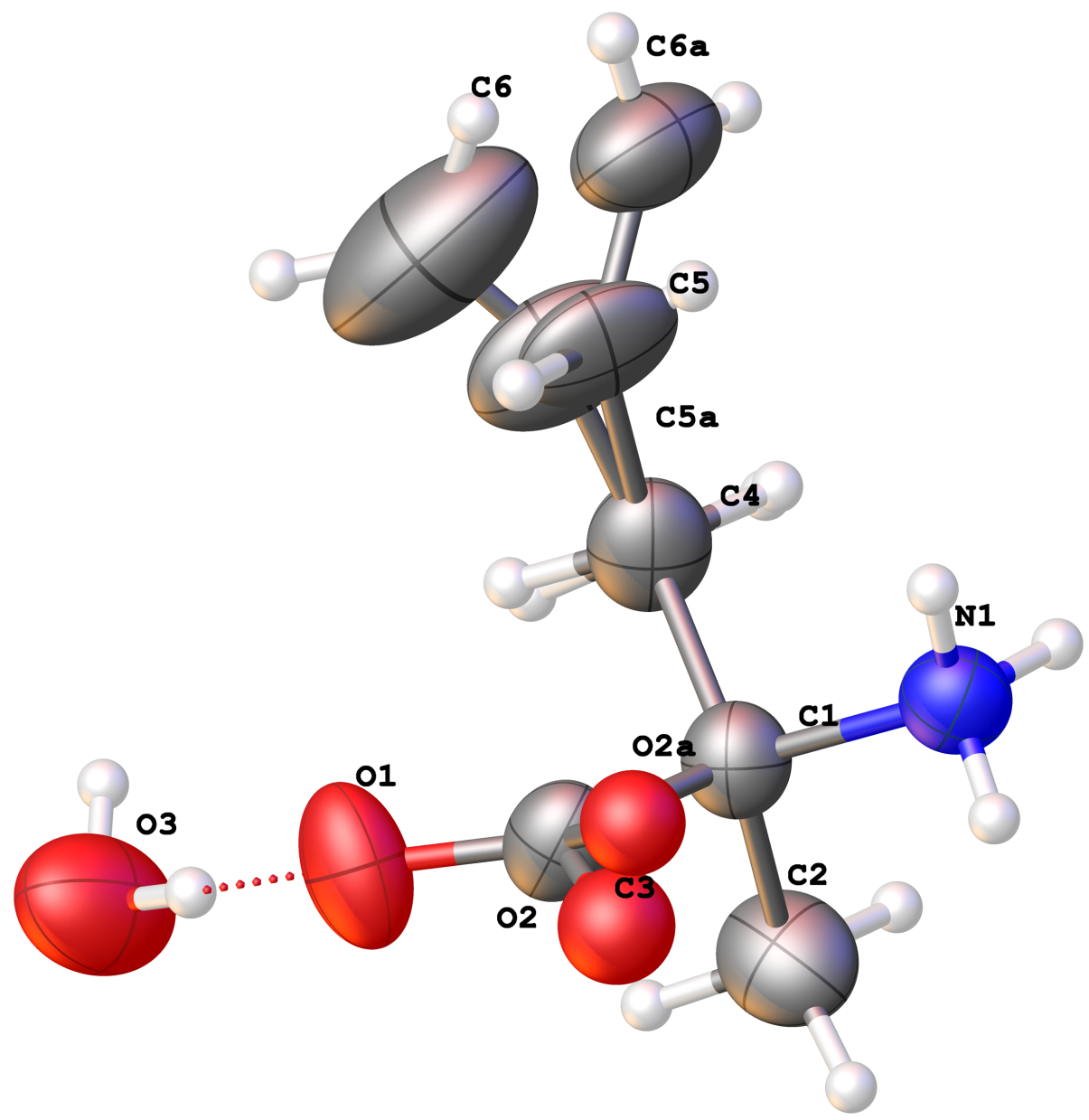
C6a
C6
C5
C5a
C4
N1
C1
O2a
O1
O3
C3
O2
C2

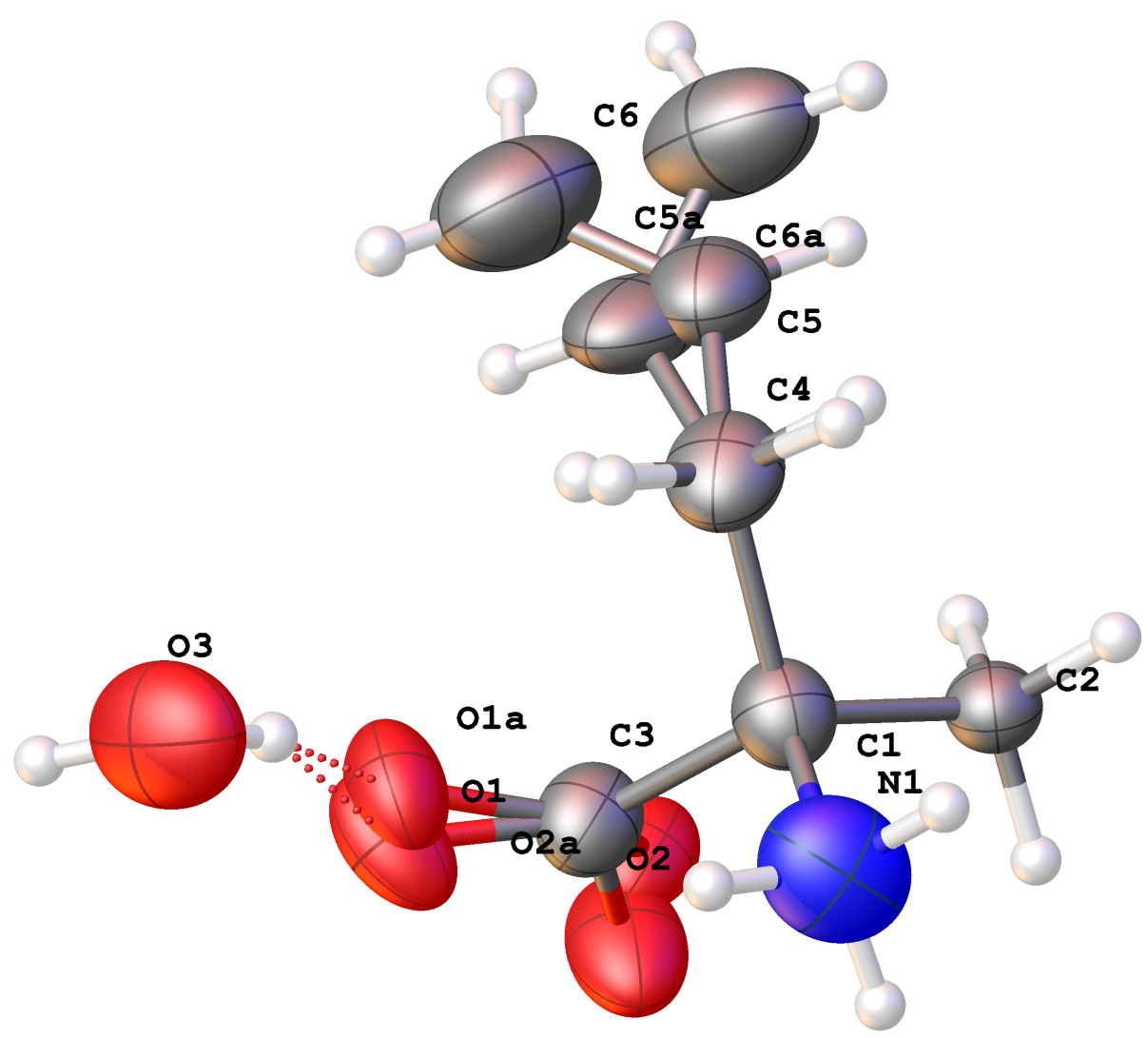
C6
C5a
C6a
C5
C4
O3
O1a
C3
C2
C1
N1
O1
O2a
O2

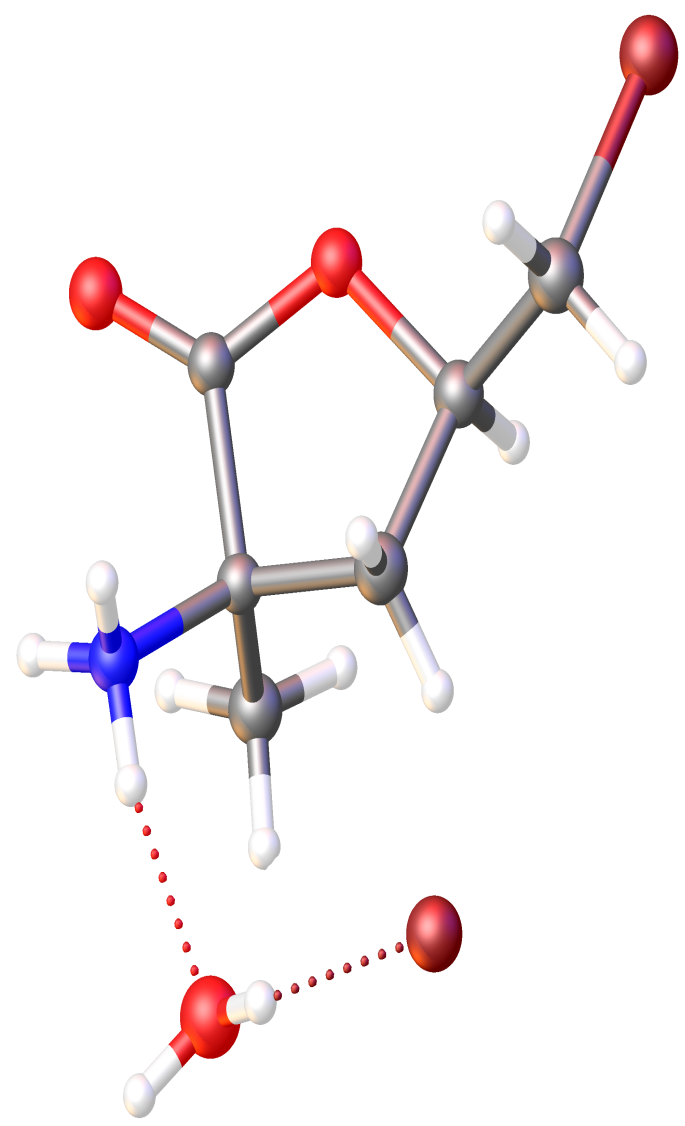

**Figure S13.** Asymmetric-unit renderings of the three single-crystal structures (rac-4; (*S*)-4; compound 10).

## S14. References

1. R. Chung and J. E. Hein, *React. Chem. Eng.*, 2019, **4**, 1674–1681.

2. M. Pattarozzi, C. Zonta, Q. B. Broxterman, B. Kaptein, R. De Zorzi, L. Randaccio, P. Scrimin and G. Licini, *Org. Lett.*, 2007, **9**, 2365–2368.
